# Flavonoids metabolism and physiological response to ultraviolet treatments in *Tetrastigma hemsleyanum* Diels et Gilg

**DOI:** 10.3389/fpls.2022.926197

**Published:** 2022-09-15

**Authors:** Yan Bai, Yiwen Gu, Shouzan Liu, Lingtai Jiang, Minqi Han, Dongjie Geng

**Affiliations:** ^1^Zhejiang Provincial Key Laboratory of Resources Protection and Innovation of Traditional Chinese Medicine, Hangzhou, Zhejiang, China; ^2^College of Food and Health, Zhejiang Agriculture and Forestry University, Hangzhou, Zhejiang, China; ^3^State Key Laboratory of Subtropical Silviculture, Zhejiang Agriculture and Forestry University, Hangzhou, Zhejiang, China; ^4^Botanical Garden, Zhejiang Agricultural and Forestry University, Zhejiang, China

**Keywords:** *Tetrastigma hemsleyanum* Diels et Gilg, flavonoid, ultraviolet radiation, metabolic expression, physiological response

## Abstract

*Tetrastigma hemsleyanum* Diels et Gilg is a folk herb in Zhejiang Province with anti-inflammatory, antineoplastic, and anti-oxidation effects. Given its pharmacological activity, *T. hemsleyanum* is known as New “Zhebawei” and included in the medical insurance system of Zhejiang and other provinces. Flavonoids are the most important components of *T. hemsleyanum*, and their contents are mainly regulated by ultraviolet (UV) radiation. In this study, the total flavonoid contents, flavonoid monomer contents, and flavonoid synthesis related enzyme activities (phenylalanine ammonia–lyase, chalcone synthase, and chalcone isomerase), anti-oxidant enzyme activities (catalase, peroxidase, and superoxide dismutase), and biochemical indicators (malondialdehyde, free amino acid, soluble protein, and soluble sugar) in the leaves (L) and root tubers (R) of *T. hemsleyanum* with UV treatments were determined. Three kinds of UV radiation (UV-A, UV-B, and UV-C) and six kinds of radiation durations (15 and 30 min, 1, 2, 3, and 5 h) were used. Appropriate doses of UV-B and UV-C radiation (30 min to 3 h) induced eustress, which contributed to the accumulation of flavonoids and improve protective enzyme system activities and bioactive compound contents. Especially, certain results were observed in several special structures of the flavonoid monomer: quercetin contents in L increased by nearly 20 times, isoquercitrin contents in R increased by nearly 34 times; most of flavonoids with glycoside content, such as quercitrin (19 times), baicalin (16 times), and apigenin-7G (13 times), increased multiple times. Compared with the CK group, the flavonoid synthase activities, anti-oxidant enzyme activities, and biochemical substance contents in L and R all increased with UV treatments. This study provides a theoretical foundation for regulating flavonoids by light factors and improving the quality of *T. hemsleyanum* in production and medical industries.

## Introduction

*Tetrastigma hemsleyanum* Diels et Gilg is one of the folk herbs in Zhejiang Province; it mainly grows in mountains and under woods and is distributed in Zhejiang, Fujian, Guangxi, etc., in China. According to ancient records, *T. hemsleyanum* is widely used in folk herbs and treatment of children with high fever, convulsion, and dysentery (Ji et al., 2021); thus, it is also called a plant antibiotic. Modern studies showed that *T. hemsleyanum* has anti-inflammatory, antineoplastic, and anti-oxidant effects (Ye and Liu, 2015; Chen et al., 2019) and is widely used in clinical practice. Given its powerful pharmacological activity, *T. hemsleyanum* is known as New “Zhebawei” and included in the medical insurance system of Zhejiang and other provinces. In addition, *T. hemsleyanum* has been used as the main raw material to produce Chinese patent medicines with tumor prevention and treatment effects and the main material in the anti-COVID-19 prescription (He et al., 2020; Zhang et al., 2022) in Zhejiang Province.

The main components of *T. hemsleyanum* are flavonoids, terpenoids, steroids, polysaccharides, and phenolic acids, among which flavonoids are the most important (Liu et al., 2022). To date, numerous flavonoid compounds have been found in *T. hemsleyanum* (Zhu et al., 2020); these compounds include kaempferol, isoquercetin, polygonin, and vitexin, and they have important functions. Flavonoids are ubiquitous functional factors in plants, showing a variety of biological activities, including anti-oxidant (Sakina et al., 2015), anti-inflammatory (Zaragozá et al., 2020), and immunomodulatory (Jasso-Miranda et al., 2019) effects. Numerous studies (Maan et al., 2020; Bondonno et al., 2021) found the correlation between flavonoid consumption and reduced incidence of diseases, such as diabetes, asthma, hypertension, cardiovascular disease, age-related diseases, and neurodegenerative diseases (such as Parkinson’s and Alzheimer’s).

Therefore, finding a way to increase the flavonoid contents in *T. hemsleyanum* has drawn our attention. At present, the common methods for increasing flavonoid contents are stress treatments, including ultraviolet (UV) (Rao et al., 2019), oxidative (Nakabayashi et al., 2014), and drought stresses (Davies et al., 2018). UV radiation constitutes 7% of all sunlight that reaches the Earth (Müller-Xing et al., 2014). UV-A (320–400 nm), UV-B (280–320 nm), and UV-C (100–280 nm) are the three primary regions of the UV spectrum, which extends from 100 to 400 nm. Rao et al. (2019) reported that one of the most important mechanisms of plants exposed to radiation to resist damage is their reliance on flavonoids (flavonoids, flavonols, isoflavones, and anthocyanins), which play a protective role by providing a strong antioxidant capacity. UV radiation can boost the accumulation of flavonoids in numerous plants. Szopa et al. (2018) showed that UV-A increased the contents of rutin, luteolin, and quercetin in *Aronia melanocarpa*, *A. arbutifolia*, and *A. prunifolia*. UV-B can increase the contents of multein, oleuropein, and luteonin-7-O-glucoside in *Olea europaea* (Dias et al., 2020). Guajardo-Flores et al. (2014) discovered that UV-C treatment mainly improved the levels of quercetin-3-O-glucoside by about two times relative to those in the control group of *Phaseolus vulgaris*.

Our previous studies showed that the total flavonoid contents of forest-undergrowth *T. hemsleyanum* were higher than those in field cultivation. Given that short-wave light dominates the understory spectrum, we attempted to increase the flavonoid content of *T. hemsleyanum* with UV radiation. A limited number of studies focused on the effects of UV treatment on flavonoids in *T. hemsleyanum*. Thus, this study aimed to evaluate the effects of different UV wavelengths and doses on the accumulation of various flavonoids in *T. hemsleyanum*. On the basis of relationships among accumulated flavonoids, flavonoid synthesis related enzymes, and substances related to anti-stress under different radiation wavelengths and doses, the regulation of flavonoids by UV radiation was explored. Our results will be helpful to improve the quality of *T. hemsleyanum* and beneficial for its development and utilization in the future.

## Materials and methods

### Materials

This study was conducted in Zhejiang Provincial Key Laboratory of Resource Protection and Innovative Utilization of Traditional Chinese Medicine, the State Key Laboratory of Subtropical Forest Cultivation. *T. hemsleyanum* plants were purchased from Suichang Planting Base (Lishui City, Zhejiang Province) and identified by Dr. Aicun Zhou of Zhejiang A&F University.

All plants grew in the Pingshan Practice base (Lin’an, Zhejiang province, 30° 15′ 30.39″ N, 119° 43′ 26.92″ E) in 18 cm diameter plastic pots. Each plant was cultivated in a pot containing a soil mixture with peat:pastoral soil:perlite:cow dung ratio of 4:4:4:1.

### Ultraviolet treatment

*Tetrastigma hemsleyanum* with the same growth vigor (3 year growths, vegetative growth phase) were transplanted to the laboratory. The aboveground plant parts were treated with UV irradiation in a shelf with an opaque black cloth. After UV irradiation for 15 and 30 min and 1, 2, 3, and 5 h, all plants were treated with darkness for 24 h. Leaves (L) and root tubers (R) were harvested as experimental materials. The details are shown in Table 1. The L treated with UV-A for 15 min and dark treatment for 23 h and 45 min were denoted as L-UV-A (15 min). Three kinds of UV light (Huaqiang Electronics Co., Ltd., China) were used: 40 W UV-A (320–400 nm), UV-B (280–320 nm), and UV-C (200–280 nm). The light intensity range was 1,630–1,660 Lx. Each treatment group comprised five biological replicates.

**TABLE 1:** Ultraviolet treatment conditions.

UV kinds	UV treatment time	Dark treatment time (after UV treatment)	Tissue collected
UV-A/UV-B/UV-C	15 min	23 h 45 min	Leaves/root tubers
	30 min	23 h 30 min	
	1 h	23 h	
	2 h	22 h	
	3 h	21 h	
	5 h	19 h	

### Metabolome analysis of flavonoids

The determination of flavonoid monomers was performed as described by Bai et al. (2021). Approximately 0.1 g freeze-dried L or R were ground into fine powder (80-mesh sieve) and placed in a 2 ml Eppendorf tube. Approximately 1.5 ml 85% methanol solution was added. The solution was ultrasonicated for 30 min, cooled and stood at room temperature, shaken uniformly, and left to stand. The supernatant was filtered with 0.22 μm membranes, and the subsequent filtrate was collected as the test solution.

The optimal chromatographic conditions were ACQUITY UPLCTM I-Class (Waters, Milford, MA, United States), ACQUITY UPLC BEH C18 column (2.1 mm × 100 mm, 1.8 μm) and 40°C. The mobile phase consisted of 0.1% formic acid–water (A) and 0.1% formic acid–acetonitrile (B) with a gradient elution of 5% B (0 min), 25% B (1 min), 40% B (3.5 min), and 60% B (4.5 min). The flow rate was 0.6 ml/min, and the injection volume was 1 μl. The mass spectrometry parameters were as follows: quadrupole ion trap mass spectrometer API 6500 (AB SCIEX, Framingham, MA, United States); ion source, Turbo V; ionization mode, ESI-; curtain gas flow rate, 30 L/min; spray voltage, −4500 V; atomized gas (GS1) flow rate, 55 L/min; auxiliary gas (GS2) flow rate, 55 L/min; collection mode, multiple reaction monitoring mode; ionization temperature, 550°C.

Supplementary Table 1 shows the optimized conditional parameters. The linear relationship results are shown in Supplementary Table 2.

### Determination of total flavonoid content

Total flavonoid contents in L and R of *T. hemsleyanum* were determined by a UV–visible (UV-VIS) spectrophotometer (UNICO 3802, Shanghai, China) in accordance with the method of Bai et al. (2021). Supplementary Method 1 provides more details on the experiment.

### Determination of flavonoid synthesis related enzymes activity

The extraction method of crude enzyme solution was as follows: 0.5 g fresh L or R were ground with 9 ml buffer solution into a homogenate and centrifuged at 10,000 r/min for 20 min at 4°C, and the supernatant was used as the extract.

Phenylalanine ammonia–lyase (PAL) enzyme activity was determined following the method of Bai et al. (2021). We obtained 1 ml crude enzyme solution, 1 ml 0.33% phenylalanine solution, and 2 ml distilled water and water bathed the mixture for 30 min at 30°C. A total of 0.2 ml 6 mol/L hydrochloric acid solution was added to terminate the reaction. The absorbance was measured by a UV-VIS spectrophotometer at 290 nm.

Chalcone synthase (CHS), chalcone isomerase (CHI), flavanone 3-hydroxylase (F3H), flavonol synthase (FLS), flavone synthase (FNS), flavonoid-3′-hydroxylase (F3′H), and flavonoid-3′,5′-hydroxylase (F3′5′H) enzyme activities were determined using the CHI, CHS, F3H, FLS, FNS, F3′H, and F3′5′H kits, respectively (Wanxiang Hengyuan Co., Ltd., Tianjin, China).

### Determination of biochemical indexes

Catalase (CAT), peroxidase (POD), and superoxide dismutase (SOD) activities and malondialdehyde (MDA) contents were determined in accordance with the method described by Ma et al. (2019). More experimental details are provided in Supplementary Method 2.

The contents of free amino acid, soluble proteins, and soluble sugars were determined using the ninhydrin, Coomassie bright blue, and anthrone methods, respectively (Mao et al., 2021). More experimental details are provided in Supplementary Method 3.

### Statistical analysis

Analyses were performed with SPSS 19.0 software. The results were statistically analyzed by using one-way analysis of variance, followed by least significant difference test at a probability level of 0.05.

Multivariate principal component analysis (PCA), variable importance in projection (VIP) analysis, and correlation chart analysis were performed with MetaboAnalyst (Chong et al., 2018). A cluster heatmap was generated with TBtools software (Chen et al., 2018).

## Results

### Flavonoid monomers content in leaves and root tubers were significantly different

**FIGURE 1:**
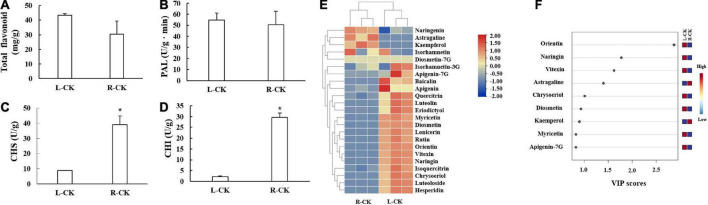
Flavonoid contents and flavonoid synthesis related enzymes activities in control group of *Tetrastigma hemsleyanum*. **(A)** Total flavonoids. **(B)** Phenylalanine ammonia-lyase (PAL). **(C)** Chalcone synthase (CHS). **(D)** Chalcone isomerase (CHI). **(E)** Heatmap of flavonoid metabolites. **(F)** VIP scores. Metabolites with VIP scores higher than 1.0 were obtained through PLS-DA. *P < 0.05.

The flavonoid contents and flavonoid synthesis related enzyme activities of *T. hemsleyanum* were detected (Figure 1). The total flavonoid contents in L were slightly higher than those in R, but the changes were not significant. As for flavonoid synthesis related enzymes, PAL enzyme activities in L and R showed no significant difference, CHS and CHI enzyme activities in R were significantly higher than those in L.

The heatmap showed that most of flavonoid monomers in L were higher than those in R (Figure 1E). Six components were identified as differential metabolites (criteria: VIP > 1.0, *t*-test *P* < 0.05). The contents of orientin, naringin, vitexin, chrysoeriol, and diosmetin in L were significantly higher than those in R, but the contents of astragaline were lower (Figure 1F).

### Content of glycosylated flavonoids increased significantly with ultraviolet treatments

**FIGURE 2:**
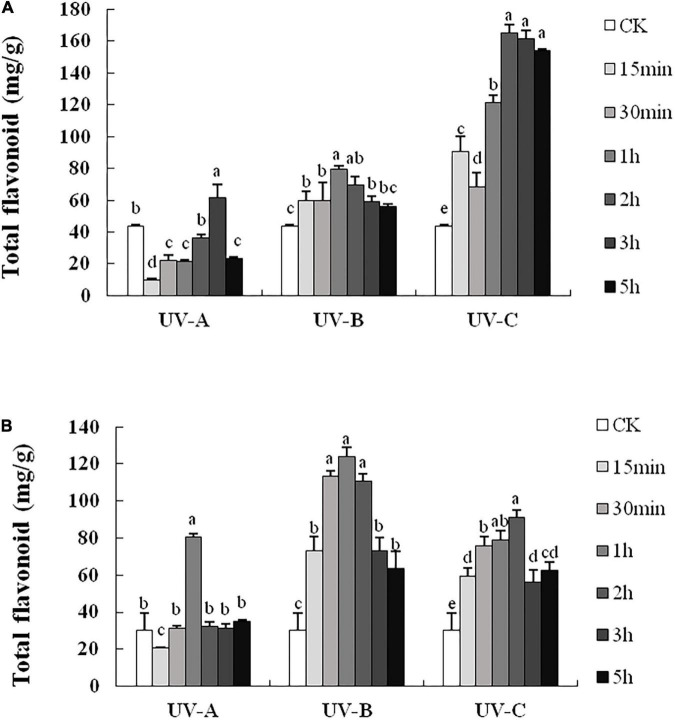
Total flavonoid contents in leaves **(A)** and root tubers **(B)** of *Tetrastigma hemsleyanum* with UV-A, UV-B, and UV-C treatments. Different lowercase letters indicate significant difference at 0.05 level (*P* < 0.05).

The flavonoid contents in L and R all increased with UV treatments. In L (Figure 2A), the total flavonoid contents with UV-C treatment were significantly higher than those in other groups, especially in the 2, 3, and 5 h groups, whose total flavonoid contents were 3.8, 3.7, and 3.5 times (164.98, 161.51, and 153.77 mg/g), respectively, those of the CK group (43.41 mg/g). Compared with the control group (1.060 mg/g), the total flavonoid contents obtained with UV-B treatment were always higher than those of other treatment groups. However, the contents of the UV-A group were lower than those of the CK group. In R (Figure 2B), the total flavonoid contents increased under UV-B exposure. Especially, the total flavonoid content of UV-B 1 h group reached the highest level (123.89 mg/g) and was considerably higher (4.1 times) than that of the CK group.

**FIGURE 3:**
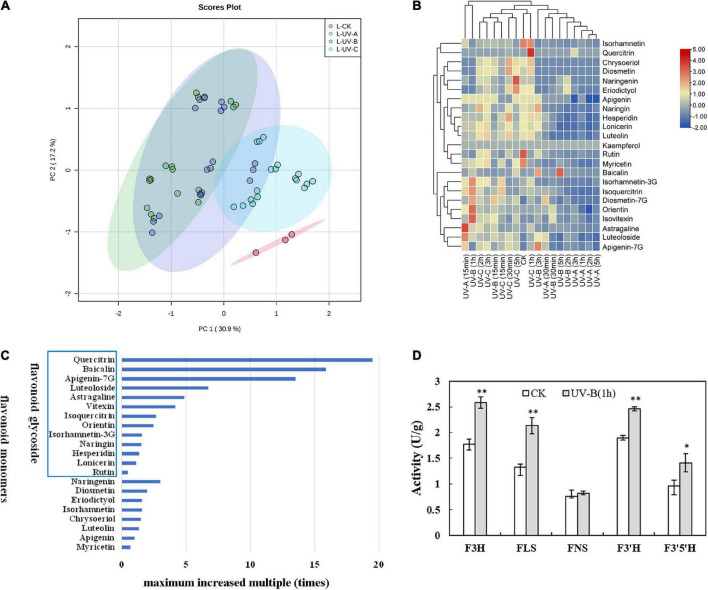
Flavonoid metabolites in leaves of *Tetrastigma hemsleyanum* with UV treatments. **(A)** PCA score plot of metabolites in leaves. **(B)** Heatmap of flavonoid metabolites in leaves. **(C)** The maximum increased multiple of flavonoid monomers with different structural characteristics in leaves. **(D)** Activities of flavanone 3-hydroxylase (F3H), flavonol synthase (FLS), flavone synthase (FNS), flavonoid-3′-hydroxylase (F3′H), flavonoid-3′,5′-hydroxylase (F3′5′H) enzyme with UV-B 1 h treatment in leaves. *P < 0.05, **P < 0.05.

**FIGURE 4:**
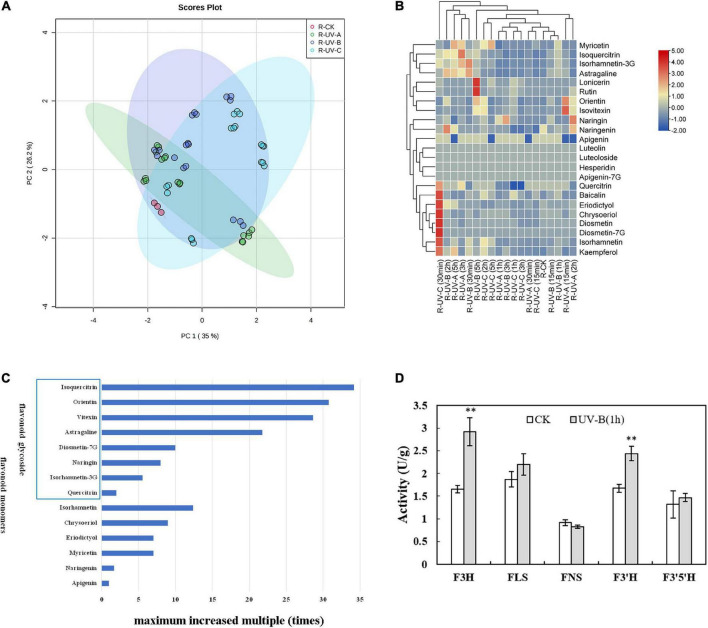
Flavonoid metabolites in root tubers of *Tetrastigma hemsleyanum* with UV treatments. **(A)** PCA score plot of metabolites in root tubers. **(B)** Heatmap of flavonoid metabolites in root tubers. **(C)** The maximum increased multiple of flavonoid monomers with different structural characteristics in root tubers. **(D)** Activities of flavanone 3-hydroxylase (F3H), flavonol synthase (FLS), flavone synthase (FNS), flavonoid-3′-hydroxylase (F3′H), flavonoid-3′,5′-hydroxylase (F3′5′H) enzyme with UV-B 1 h treatment in root tubers. *P < 0.05, **P < 0.05.

Supplementary Tables 3, 4 show the influences of radiation wavelength, radiation type, and radiation time on the contents of different flavonoid monomers. Based on targeted metabolomics, PCA was performed to evaluate the variations in the levels of flavonoid metabolites. The PCA score plot results indicated that the effect of UV radiation on L was more evident than that on R. In L (Figure 3A), the CK group was separated from the other treatment groups. Especially, the UV-C group was significantly separated with other groups, and the UV-A group was located close to the UV-B group. In R (Figure 4A), four groups were located close together and could not be separated. In addition, the effect of UV-C treatment on flavonoids in L was different from those of UV-A and UV-B.

Heatmap analysis provided an integrated view of UV radiation effect on L (Figure 3B) and R (Figure 4B). In L, the flavonoid contents increased under the three UV treatments. Especially under UV-B and UV-C radiation, the flavonoid content increased compared with those in the control group. Notably, short-time (15 min) UV-A treatment increased the levels of most flavonoid monomers, especially the contents of apigenin-7G, luteoloside, astragaline, and isoquercitrin, whereas more than 30 min UV-A treatment had less effect on flavonoid accumulation. Long-time UV-B radiation reduced the flavonoid contents, but less than 1 h of radiation improved the contents of most flavonoids (isovitexin, orientin, diosmetin-7G, isoquercitrin, and isorhamnetin-3G). UV-C radiation at all durations (15 min to 5 h) promoted the enhancement of flavonoid contents (nearly all flavonoid monomers were detected in our study). In R, the flavonoid contents increased slightly with UV in general, except under certain radiation conditions. Under UV-C radiation for 30 min, the contents of isorhamnetin, baicalin, kaempferol, quercitrin, chrysoeriol, eriodictyol, diosmetin, and diosmetin-7G increased dramatically. Under UV-B radiation for 5 h, the contents of lonicerin and rutin increased significantly. In general, UV-B and UV-C treatments are optimal methods for promoting the accumulation of flavonoid contents.

Combined with the above results, the flavonoid monomers were further classified. Compared with the control group in L (Figure 3C), most of flavones with glycoside content increased multiple times. These flavones included quercitrin (19 times), baicalin (16 times), apigenin-7G (13 times), luteoloside (7 times), astragalin (5 times), vitexin (4 times), isoquercitrin (3 times), and orientin (2 times). The same phenomenon was found in R (Figure 4C). The contents of isoquercitrin (34 times), orientin (30 times), vitexin (29 times), astragalin (22 times), diosmetin-7G (10 times), naringin (8 times), and isorhamnetin-3G (6 times) were substantially higher than those in the control group.

To gain more information on the variation in the flavonoid monomer contents with UV radiation, we randomly selected the materials treated with UV-B for 1 h and determined the activities of specific enzymes involved in the increase in flavonoid metabolites in R and L. In L (Figure 3D), the F3H, FLS, F3′H, and F3′5′H activities were significantly higher than those of the control group. In R (Figure 4D), F3H and F3′H activities were significantly higher than those of the control group.

### Activities of flavonoid synthesis related enzymes in the leaves and root tubers all increased with ultraviolet treatments

**FIGURE 5:**
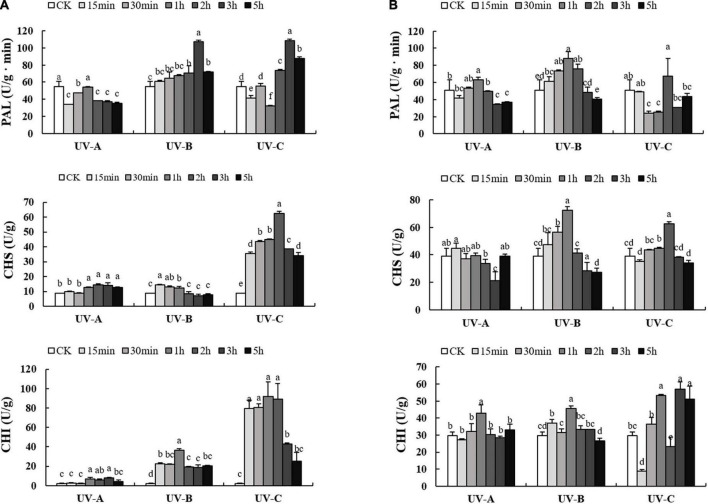
Activities of phenylalanine ammonia-lyase (PAL), chalcone synthase (CHS), chalcone isomerase (CHI) enzyme in leaves **(A)** and root tubers **(B)** of *Tetrastigma hemsleyanum* with UV-A, UV-B, and UV-C treatments. Different lowercase letters indicate significant difference at 0.05 level (*P* < 0.05).

As presented in Figure 5, the activities of flavonoid synthesis related enzymes in L and R increased with UV treatments first and then decreased as the radiation time increased. UV-A radiation reduced PAL enzyme activities at all time points in L (Figure 5A). Under UV-B or UV-C conditions, PAL enzyme activities were higher than those of the CK group, especially at UV-C 3 h (108.43 U/g min). In terms of CHS and CHI, UV-C treatments increased sharply the enzyme activities, which reached the highest values (62.55 and 89.06 U/g min, respectively) at UV-C 2 h. In R (Figure 5B), UV-B and UV-C treatments resulted in evident enzyme activities. Under UV-B exposure for 1 h, PAL and CHS enzyme activities reached the highest values (87.90 and 72.23 U/g min, respectively). Under UV-C exposure 3 h, CHI enzyme activities reached the highest value (56.83 U/g min).

### Most of substances related to anti-stress first increased and then decreased with radiation time

**FIGURE 6:**
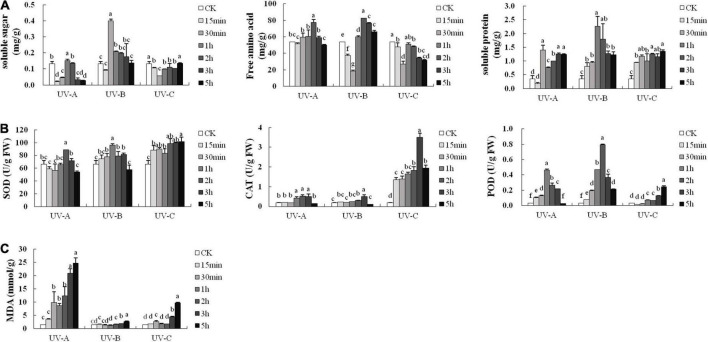
Soluble substance contents **(A)**, antioxidant enzyme activities **(B)**, and malondialdehyde (MDA) contents **(C)** of leaves in *Tetrastigma hemsleyanum* with UV treatments. Different lowercase letters indicate a significant difference at 0.05 level (*P* < 0.05).

Soluble substances are the basic nutrients in plants and can reflect plant growth and development status. Most of the soluble substance increased first and then decreased with stress duration (Figure 6A). Compared with the control group, the UV-B treatments significantly increased the total soluble sugar contents. UV-A and UV-B treatments increased the soluble amino acid contents under various treatment periods, whereas the soluble amino acid contents in the UV-C treatment group were lower than that in the CK group in each treatment period. UV-A, UV-B, and UV-C radiation increased the soluble protein content in each treatment group. The highest levels of soluble amino acid, protein, and sugar were obtained with UV-B treatments, with values of 82.74 (2 h), 2.26 (1 h), and 0.40 mg/g (30 min), respectively.

Superoxide dismutase, CAT, and POD are the main members of the protective enzyme system. Figure 6B shows the activities of these enzymes with UV treatment. UV-B and UV-C radiation improved their protective activities. The highest values of SOD, CAT, and POD activities were 101.85 (UV-C 5 h), 3.50 (UV-C 3 h), and 0.793 U/g (UV-B 2 h), respectively, which were considerably higher than those of the control group. These results indicated that L and R are in adversity under UV exposure.

Figure 6C shows that the MDA contents were maintained at low levels and close to that of the CK group with UV-B treatment at different times. The MDA contents exhibited minimal changes when the UV-C radiation time was less than 2 h. Compared with the MDA contents in UV-B and UV-C groups, those of the UV-A groups showed an upward trend and reached a maximum value (24.71 nmol/g FW) with 5 h exposure.

*Tetrastigma hemsleyanum* is a known source of phytotherapeutics. As an edible plant, the R can be used for medicinal purposes, whereas the L are consumed as functional tea or dietary supplement because of their health benefits. An increasing number of studies on *T. hemsleyanum* have been published in recent years, and its flavonoids have attracted considerable interest. Fan et al. (2017) identified orientin, vitexin, isovitexin, etc., and eight kinds of flavonoid monomers from the L of *T. hemsleyanum*. Zhu et al. (2020) reported more than 30 kinds of flavonoid monomers from whole plants (*T. hemsleyanum*). Most of these substances were detected in our study. A total of 21 flavonoids were detected in the L of *T. hemsleyanum*, and 15 flavonoids were detected in the R (Figure 1E). Shi et al. (2022) observed that the flavonoids in *T. hemsleyanum* were affected by external environment, similar to our results. We employed UV treatments to regulate the flavonoids of *T. hemsleyanum* (naringenin, astragaline, kaempferol, isorhamnetin, diosmetin-7G, isorhamnetin-3G, apigenin-7G, baicalin, apigenin, quercitrin, luteolin, eriodictyol, myricetin, diosmetin, lonicerin, rutin, orientin, vitexin, naringin, isoquercitrin, chrysoeriol, luteoloside, and hesperidin) and increased the flavonoid contents and varieties to further improve the quality of *T. hemsleyanum* (Figure 1E).

Based on the research results and analysis, UV radiation had various effects on flavonoid monomers. Several studies indicated that flavonoid monomers with diverse structures have different levels of sensitivity to UV radiation, especially those with specific structures (Murai et al., 2009). Under UV exposure, the contents of flavanol and flavonoid subclasses increased more than that of the dihydroflavonoid subclass in L and R, particularly the flavanol subclass. Zoratti et al. (2014) suggested that the flavanol subclass is the main group of flavonoids induced by UV exposure. When the flavanol subclass possessed an ortho-dihydroxy substitution at the B-ring (hydroxyl groups at C3′ and C4′), quercetin (flavonol) contents in L increased nearly 20 times with UV-C exposure for 1 h (Figure 3C). The isoquercitrin contents in R increased nearly 34 times under UV-A exposure for 1 h (Figure 4C). Numerous studies (Ryan et al., 1998; Agati et al., 2011) demonstrated that ortho-dihydroxylated B-ring flavonoids, such as quercetin and its derivatives, accumulated in plants with UV treatment. In addition, the hydroxyl groups of flavonoids exhibited an anti-oxidant activity *in vitro* and *in vivo*, promoting the radical scavenging potential (Rice-Evans et al., 1996).

Ultraviolet radiation particularly increased the content of glycoside flavonoids. Recently, abundant studies indicated that UV radiation promotes the accumulation of flavonoid glycosides in plants, especially certain functional food raw materials. In *Rhodiola rosea* L. (Dong et al., 2020), flavonoids (characterized by glycosides) were significantly up-regulated with the increasement of elevation. In *Astragalus membranaceus* (Liu et al., 2018), the contents of isoflavones, especially flavonoid glycosides, increased under UV-B radiation. In *Glycyrrhiza uralensis* (Zhang et al., 2018), the contents of glycosides in L increased under UV-B radiation. Flavonoids with glycoside perform numerous functions in plants. Modifications provide extra structural stability to flavonoids during storage in vacuoles and chloroplasts (Marinova et al., 2007). These substances confer structural complexity, molecular solubility and stability, subcellular transportability, and biological activity, which play important roles in plant growth, hormone balance, and elimination of toxicity of endogenous and exogenous substances (Xin et al., 2017). Furthermore, flavonoids with glycosides strongly enhance water solubility and thus increase bioavailability, improving anti-oxidation and exerting beneficial effects on human health (Hollman et al., 2000; Lewandowska et al., 2013).

The accumulations of these flavonoid compounds are closely related to the activation of enzymes. PAL, CHS, and CHI play important roles in flavonoid synthesis. UV exposure enhances PAL, CHS, and CHI activities, particularly in *Brassica oleracea* (Lee et al., 2019) and *Dendrobium officinale* (Chen Y. et al., 2020). Our results indicated similar effects (Figure 5), that is, significant positive correlations were found among total flavonoid content, partial flavonoid monomers, and enzyme activities. Notably, flavonoid monomer contents barely increased upstream of the pathway (naringin, apigenin, and eriodictyol). We observed that numerous flavonoid monomers were secondary metabolite products but consumed as upstream substrates simultaneously. This high consumption was probably caused by a large increase in the glycoside flavonoid content. Several studies (Wu et al., 2017; Zhang et al., 2018) showed that specific downstream flavonoid synthetases, such as FLS and UGT(), are highly sensitive to UV treatment, which further explains the increased contents of downstream flavonoids. Referring to metabolic pathways in other studies (Zhang et al., 2017; Xu et al., 2019), a close relationship was observed between the increase in eriodictyol, kaempferol, quercitrin, and luteoloside contents in *Zea mays* subsp. *mays*, *Fagopyrum tataricum*, and activities of flavonoid synthesis related enzymes, including F3H, FLS, F3’H, F3’5’H, and FNS. Correlation analysis (Supplementary Figure 1) showed that these five enzymes were significantly positively correlated with lonicerin, diosmetin-7G, isorhamnetin, quercitrin, orientin, vitexin, isoquercitrin, astragaline, luteoloside, and baicalin in the L of *T. hemsleyanum*. This finding prompted us to focus on these enzymes. We will explore further the relationship between flavonoid synthase activity and flavonoid metabolome regulated by UV treatment.

The synthesis of flavonoids is a complicated process, and although various studies have shown that flavonoid content is related to enzyme activities, a wide range of other factors affect this process. According to our results, CAT and SOD and most soluble substances have a significant positive correlation with the total flavonoid content, whereas a significant negative correlation was observed among MDA content (lipid peroxidation), the content of total flavonoids, and 70% of flavonoid monomers. In addition, soluble substance contents and protective enzyme activities showed an upward and subsequent downward trend (Figure 6A). Plants synthesize a large number of soluble substances (soluble sugars, amino acids, and proteins) with short-term UV radiation and provide basic substances for plant metabolism and osmotic adjustment to protect macromolecules and membranes. Soluble substances are the basic nutrients in plants and can reflect plant growth and development status (Ibrahim et al., 2020). Soluble sugars in plants include glucose, fructose, sucrose, and other monosaccharides and oligosaccharides (Malejane et al., 2018). Soluble amino acids comprise alanine, arginine, asparagine, and about 20 kinds of amino acids (Sun et al., 2017). Soluble proteins are proteins that can be dissolved in water or other solvents as small molecules. A variety of plant studies (Chen L. et al., 2020; Wang et al., 2022) have shown that under adverse conditions, accumulated soluble substances can regulate cell fluid concentration to regulate osmotic pressure, indicating stress resistance. In this study, we aimed to determine the state of *T. hemsleyanum* under UV stress by measuring these three indicators. The flavonoid contents must be increased through UV stress to improve the quality of *T. hemsleyanum*. We also aimed to maintain its normal growth state while giving stress. The soluble substances mentioned are very important for the growth of *T. hemsleyanum* and serve as substrates for the generation of secondary metabolites (including flavonoids). However, the correlation between flavonoid content and these substances has not been explored. Meanwhile, SOD, CAT, and POD can remove intracellular reactive oxygen species (ROS) and free radicals. SOD is the first defense line against ROS and converts highly reactive O^2–^ into less toxic H_2_O_2_ and O_2_, whereas CAT and POD convert H_2_O_2_ into H_2_O (Jing et al., 2020). As the final decomposition product of lipid peroxidation and due to the combined mechanisms above, MDA contents reflect the lipid peroxidation level and cellular lipid peroxidation damage to a certain extent (Zeng et al., 2019). Our results were similar to those of other research (Tripathi et al., 2021), short-term UV radiation significantly enhanced the anti-oxidant enzyme systems. Other studies (Li et al., 2019) showed that with further stress, the peroxidation of the membrane system was enhanced, and the MDA content increased. The MDA content can be used as an indicator to assess the resistance. Similar to our results, *T. hemsleyanum* was irreversibly damaged, and MDA content increased continuously with radiation dose (Figure 6C).

## Conclusion

Ultraviolet exposure with different durations or wavelengths can promote the increase in the contents of flavonoids with different structures, which is beneficial to the accurate regulation of special flavonoids in *T. hemsleyanum*. Appropriate UV-B and UV-C radiation doses (30 min to 3 h) can induce eustress, which can improve bioactive compound contents and protective enzyme system activities for the regulation of physiological state and enhancement of flavonoid accumulations.

## Data availability statement

The raw data supporting the conclusions of this article will be made available by the authors, without undue reservation.

## Author contributions

YB and YG contributed equally to this work and contributed to the writing of manuscript. YB designed the research and revised the manuscript. YG, SL, LJ, MH, and DG performed the experiments and disposal the data. All authors contributed to the article and approved the submitted version.
