# Supplementary figures and images for: Flavonoids metabolism and physiological response to ultraviolet treatments in Tetrastigma hemsleyanum Diels et Gilg
Source: Front Plant Sci. 2022 Sep 15;13:926197. doi: 10.3389/fpls.2022.926197 (PMC9520580; doi:10.3389/fpls.2022.926197)

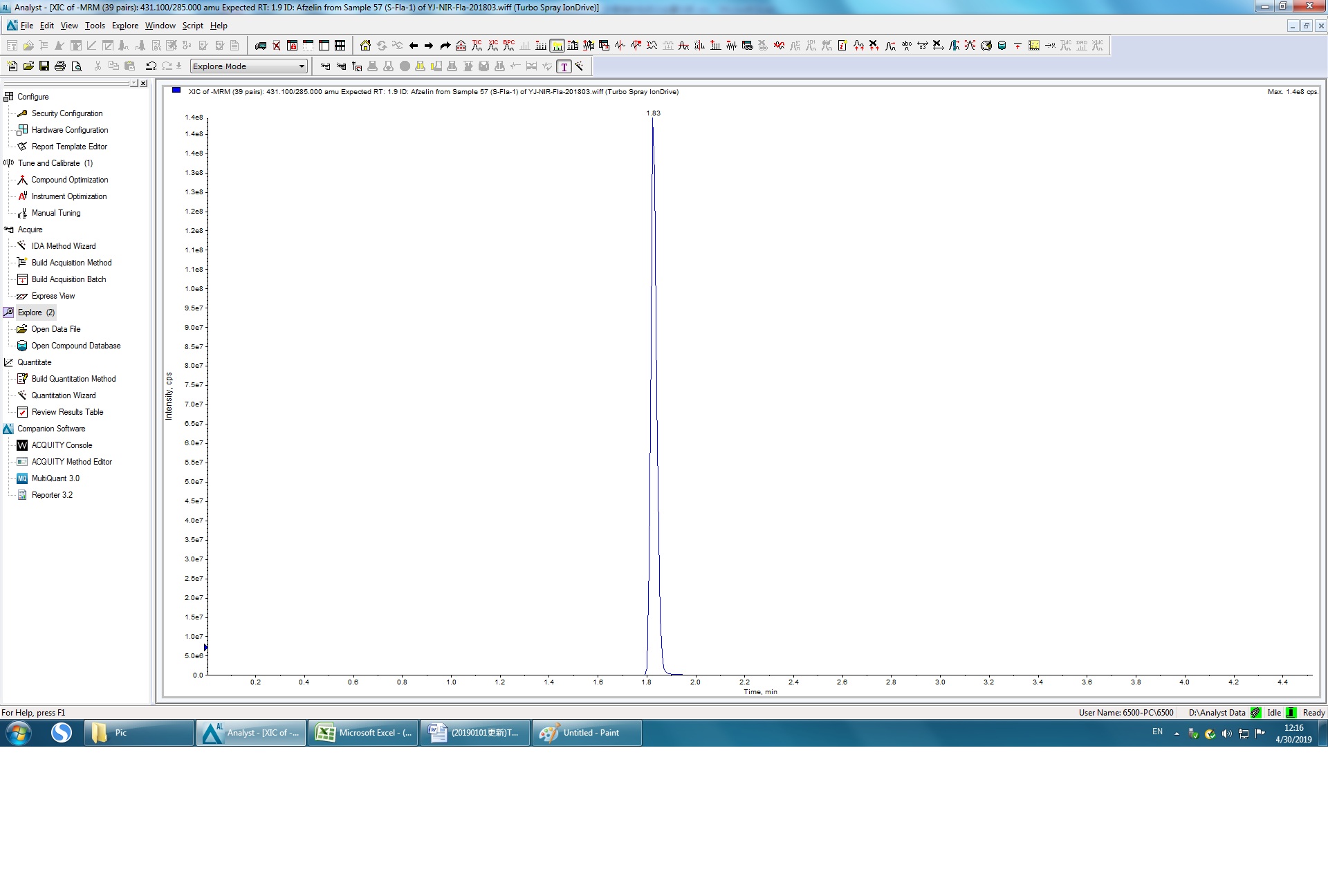

Supplement: Supplementary file 1 [file Data_Sheet_1.ZIP › Flow diagrams 926197/Afzelin.jpg]

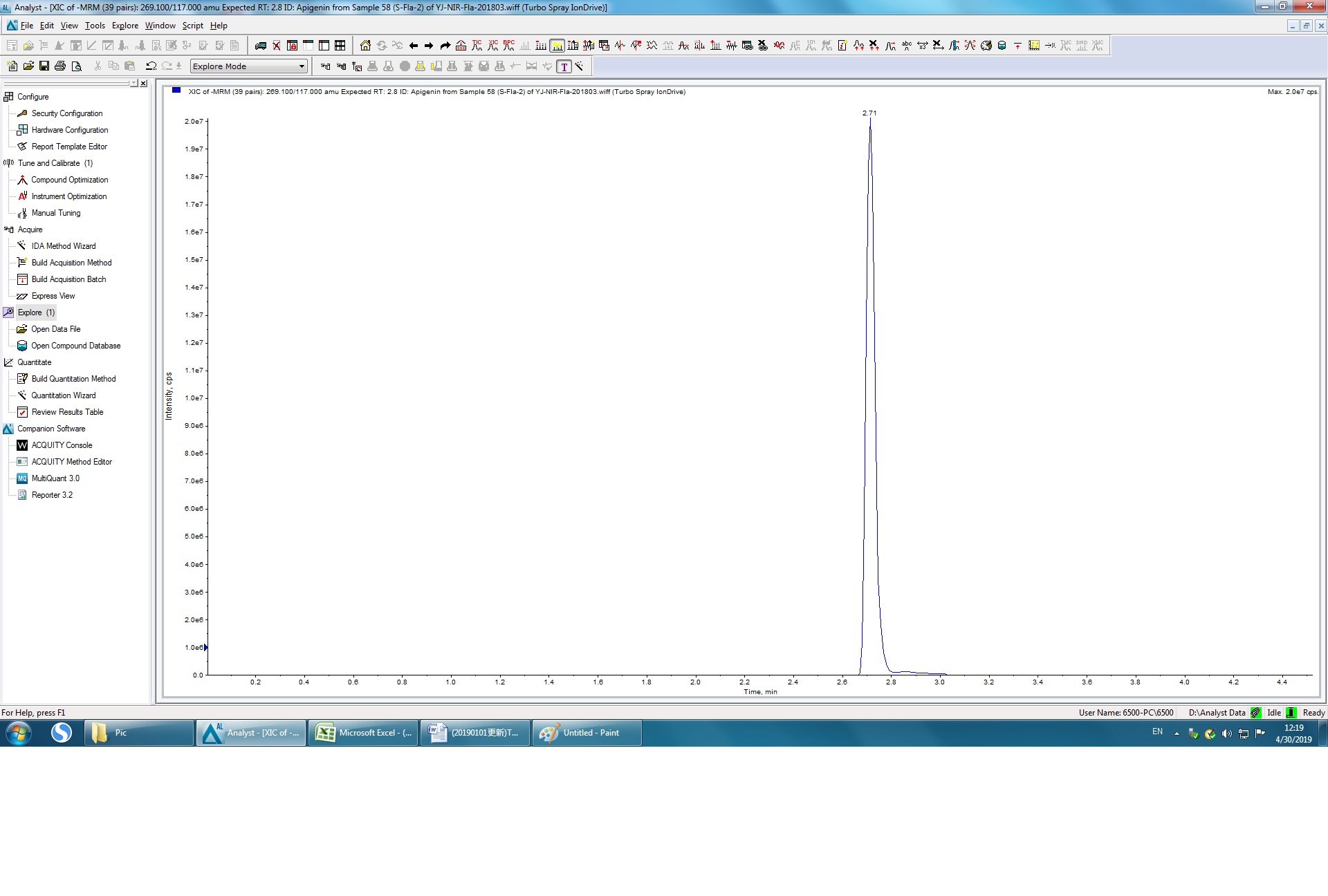

Supplement: Supplementary file 1 [file Data_Sheet_1.ZIP › Flow diagrams 926197/Apigenin.jpg]

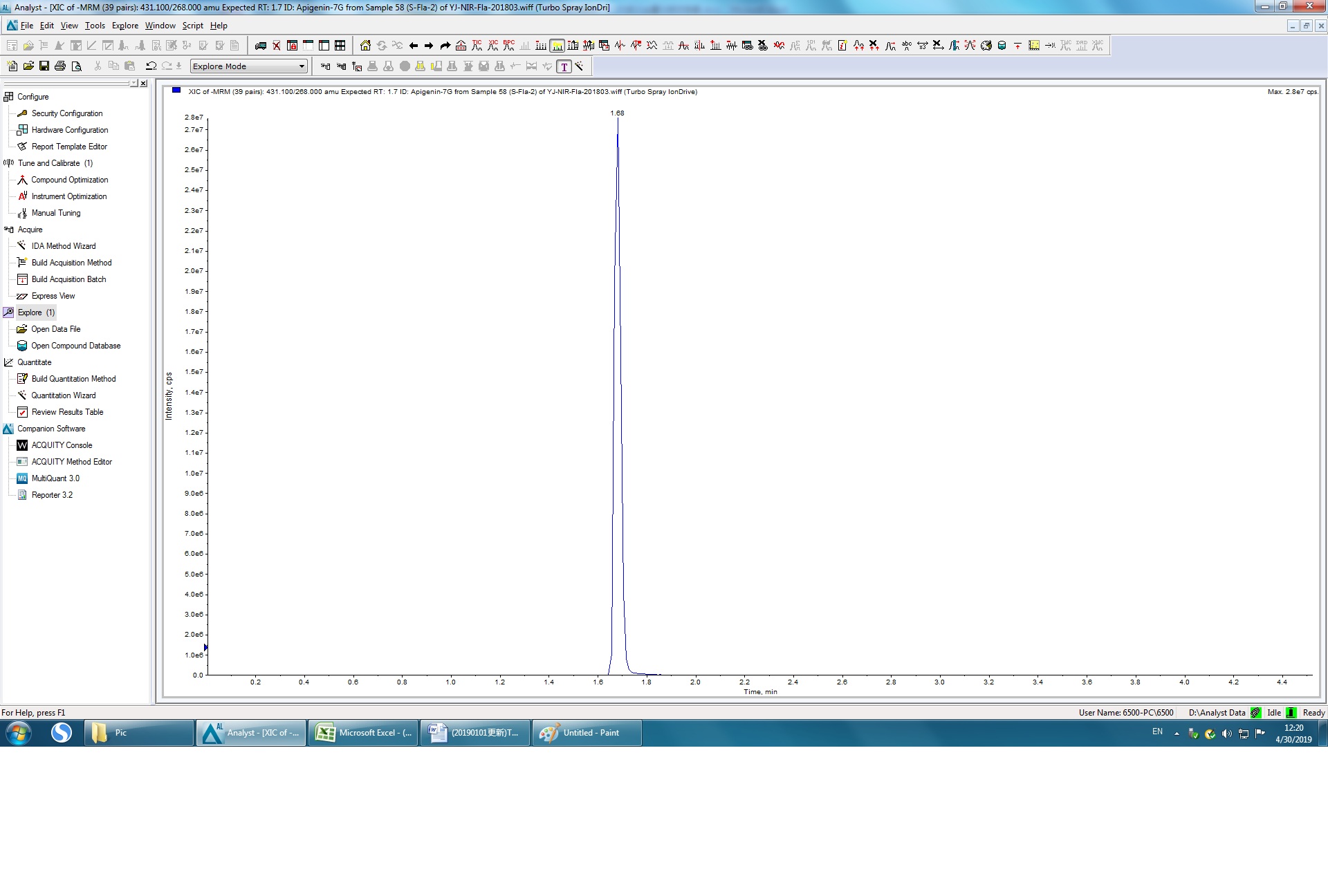

Supplement: Supplementary file 1 [file Data_Sheet_1.ZIP › Flow diagrams 926197/Apigenin7-O-glu.jpg]

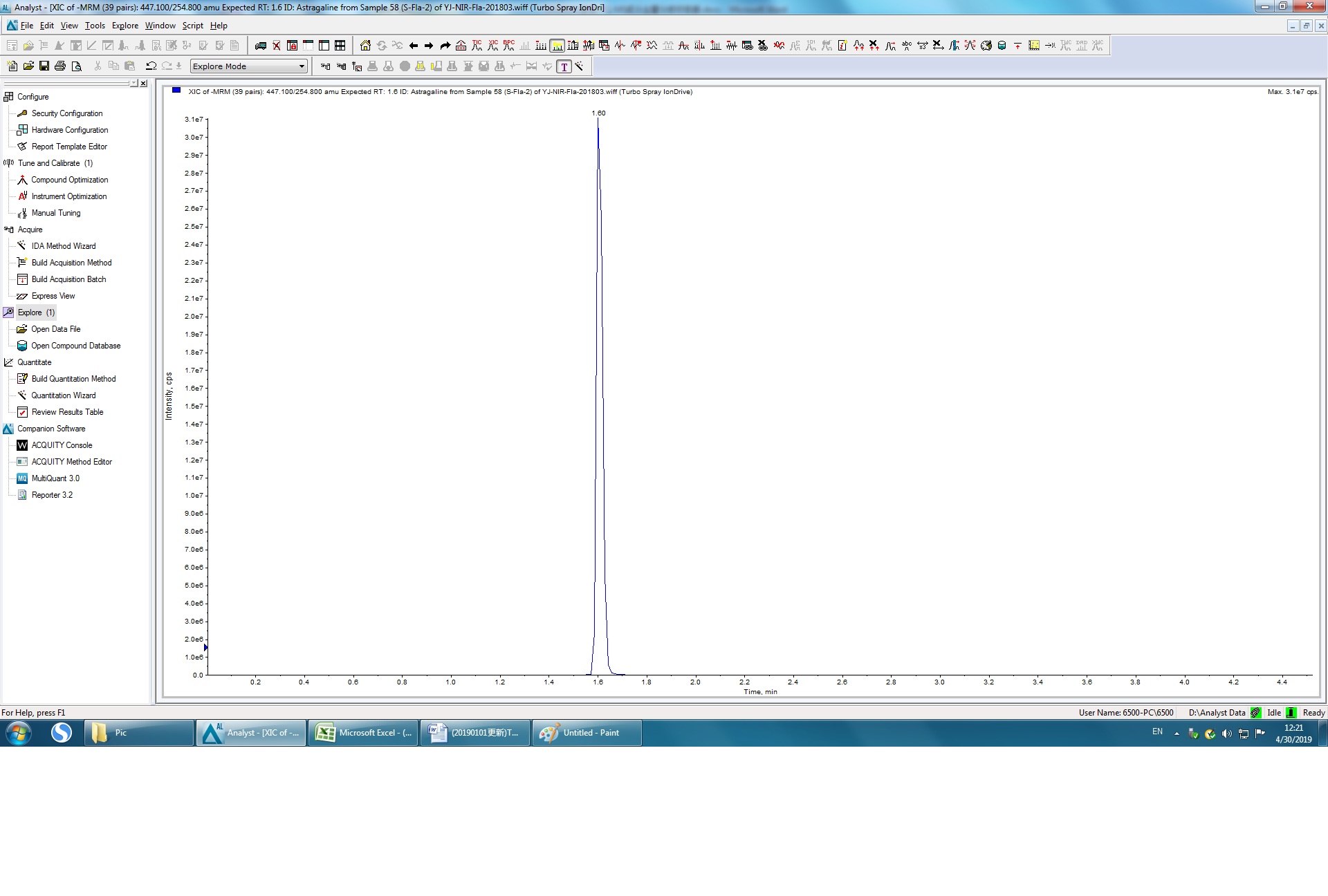

Supplement: Supplementary file 1 [file Data_Sheet_1.ZIP › Flow diagrams 926197/Astragaline.jpg]

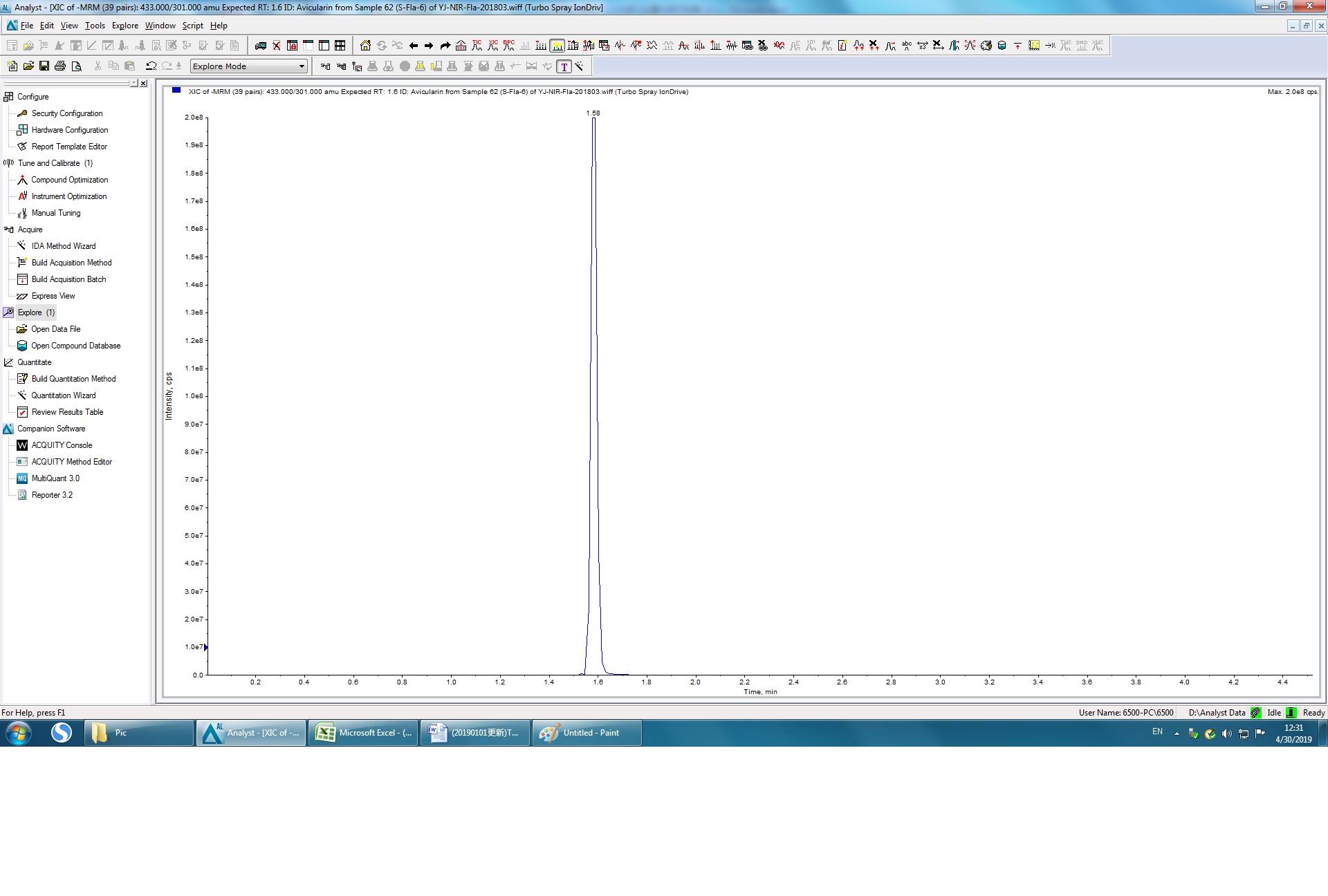

Supplement: Supplementary file 1 [file Data_Sheet_1.ZIP › Flow diagrams 926197/Avicularin.jpg]

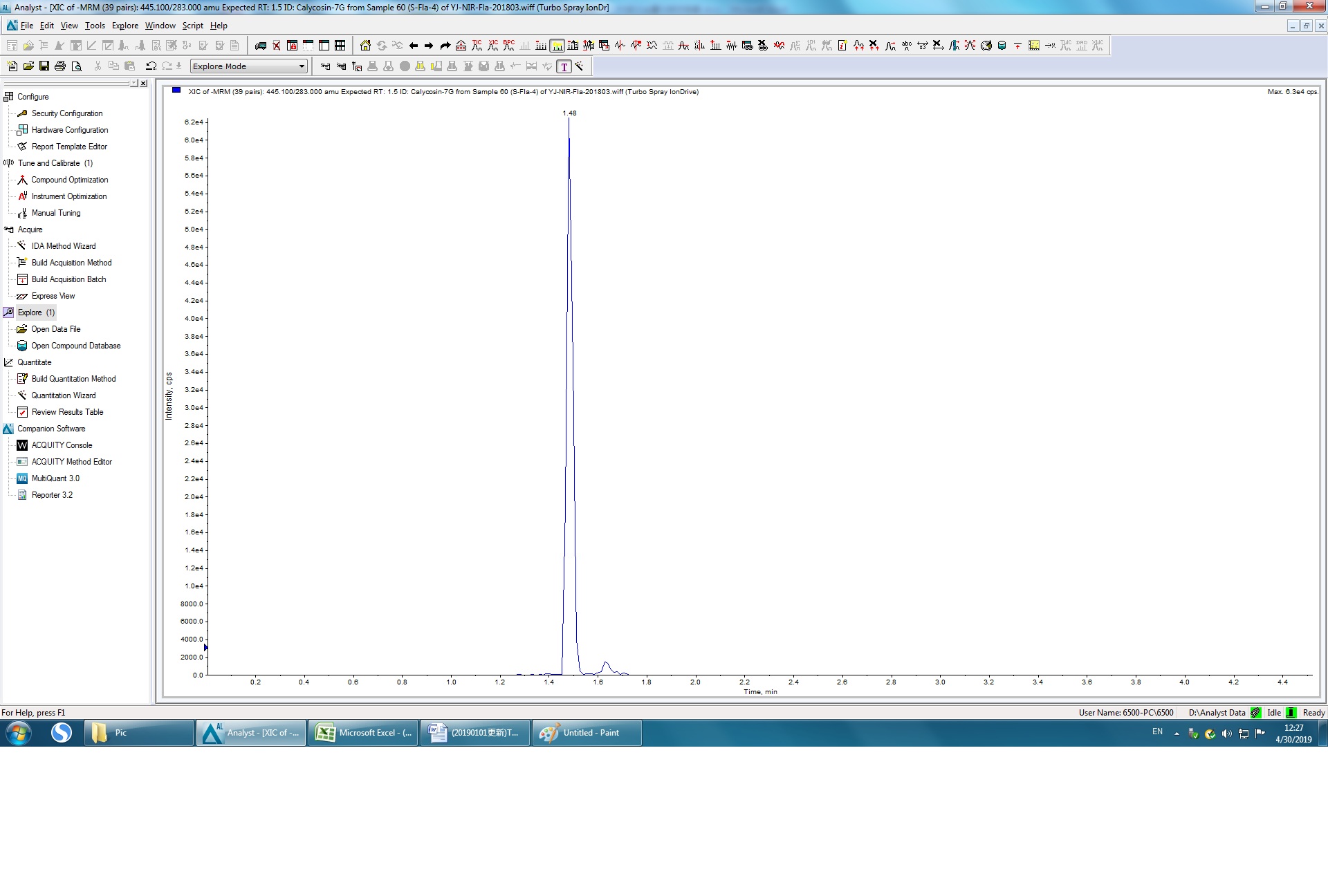

Supplement: Supplementary file 1 [file Data_Sheet_1.ZIP › Flow diagrams 926197/Calycosin 7-O-glu.jpg]

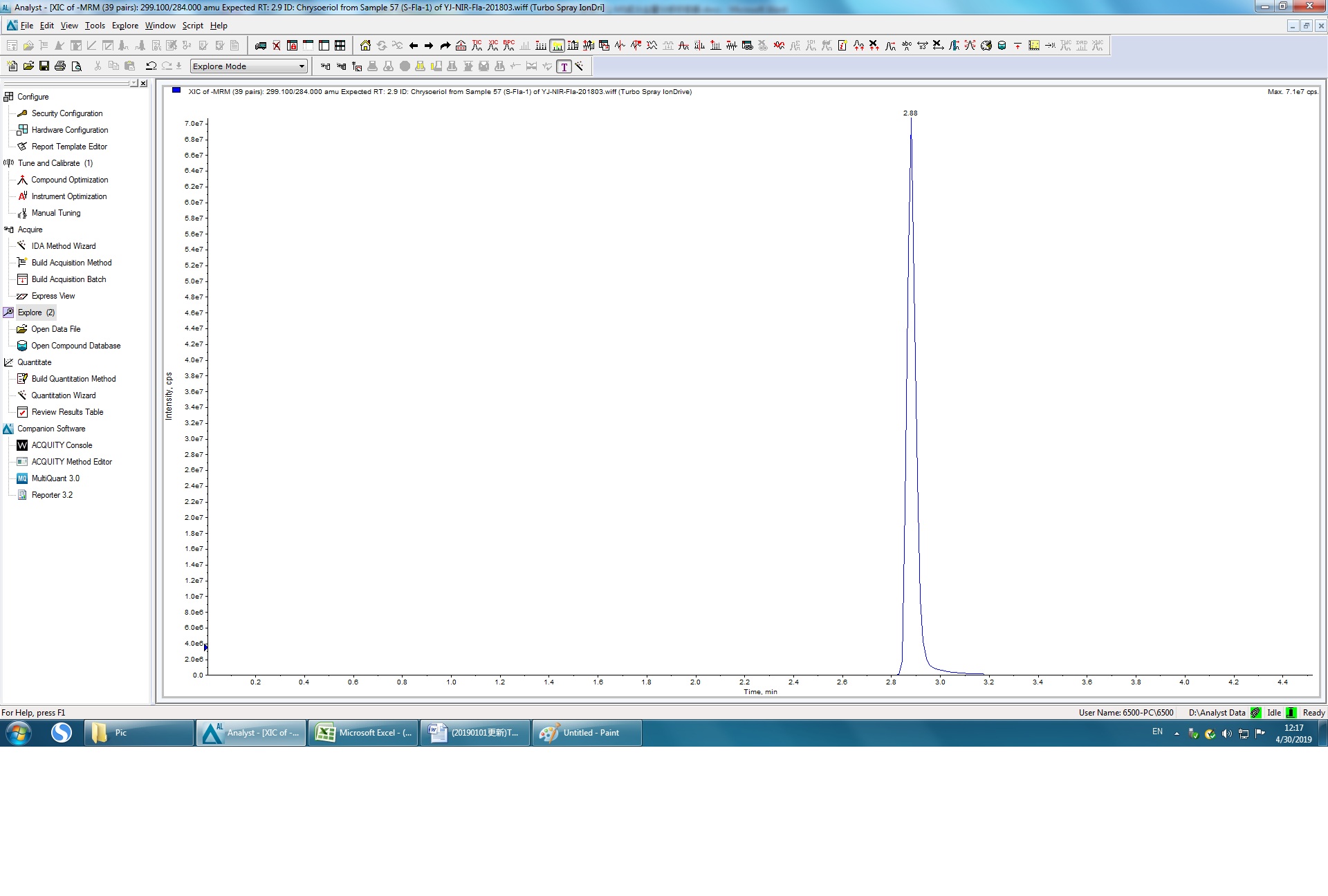

Supplement: Supplementary file 1 [file Data_Sheet_1.ZIP › Flow diagrams 926197/Chrysoeriol.jpg]

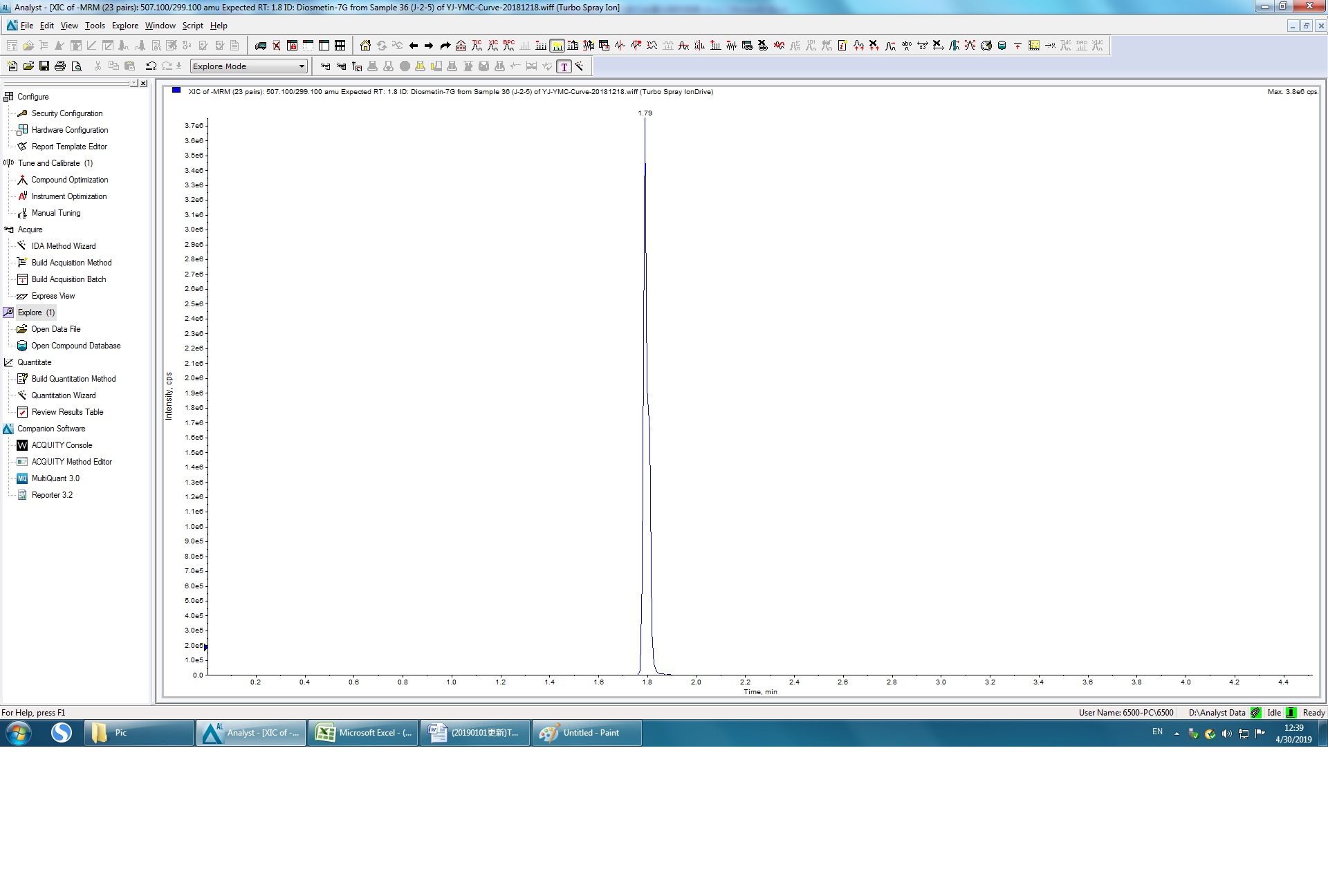

Supplement: Supplementary file 1 [file Data_Sheet_1.ZIP › Flow diagrams 926197/Diosmetin 7-O-G.jpg]

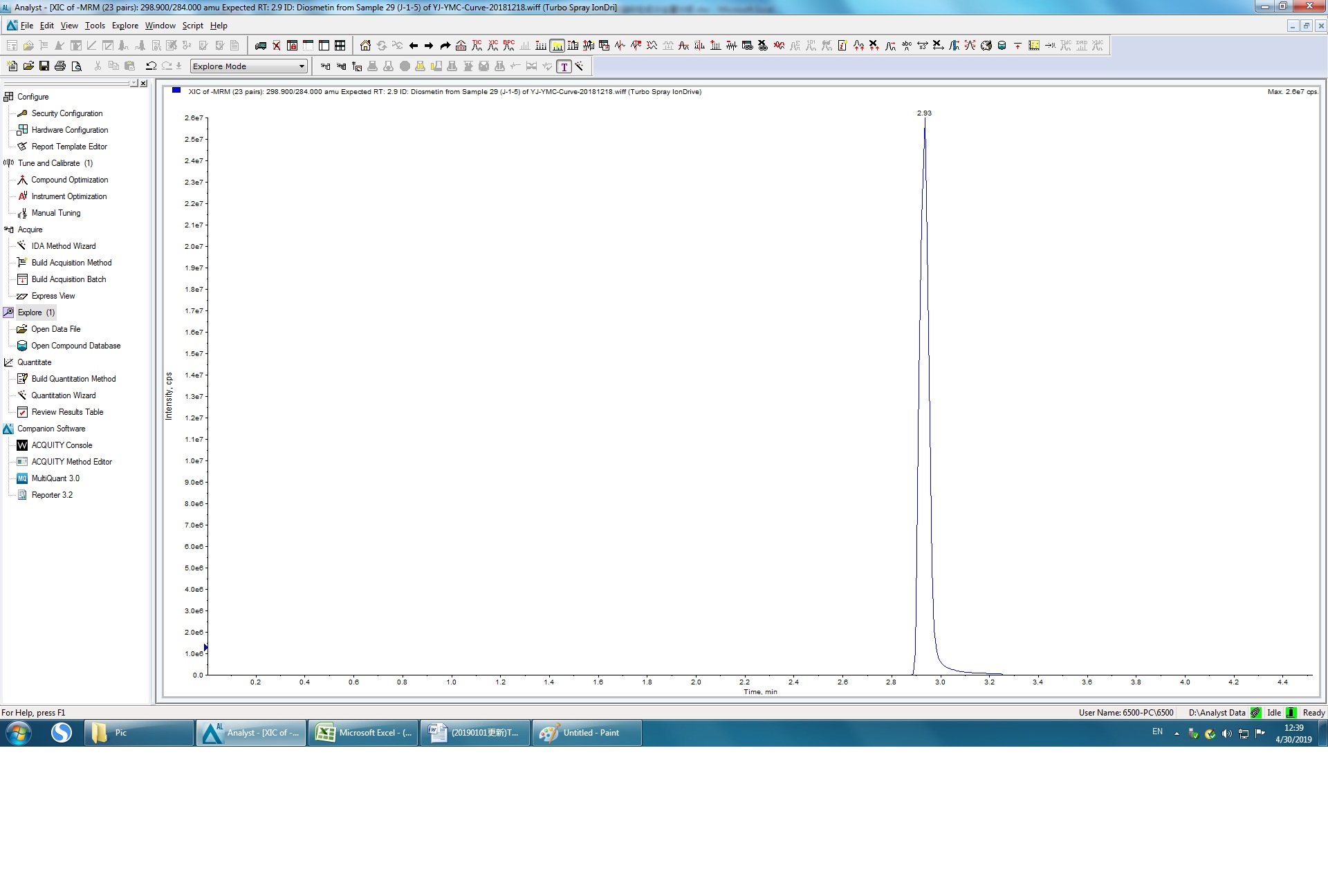

Supplement: Supplementary file 1 [file Data_Sheet_1.ZIP › Flow diagrams 926197/Diosmetin.jpg]

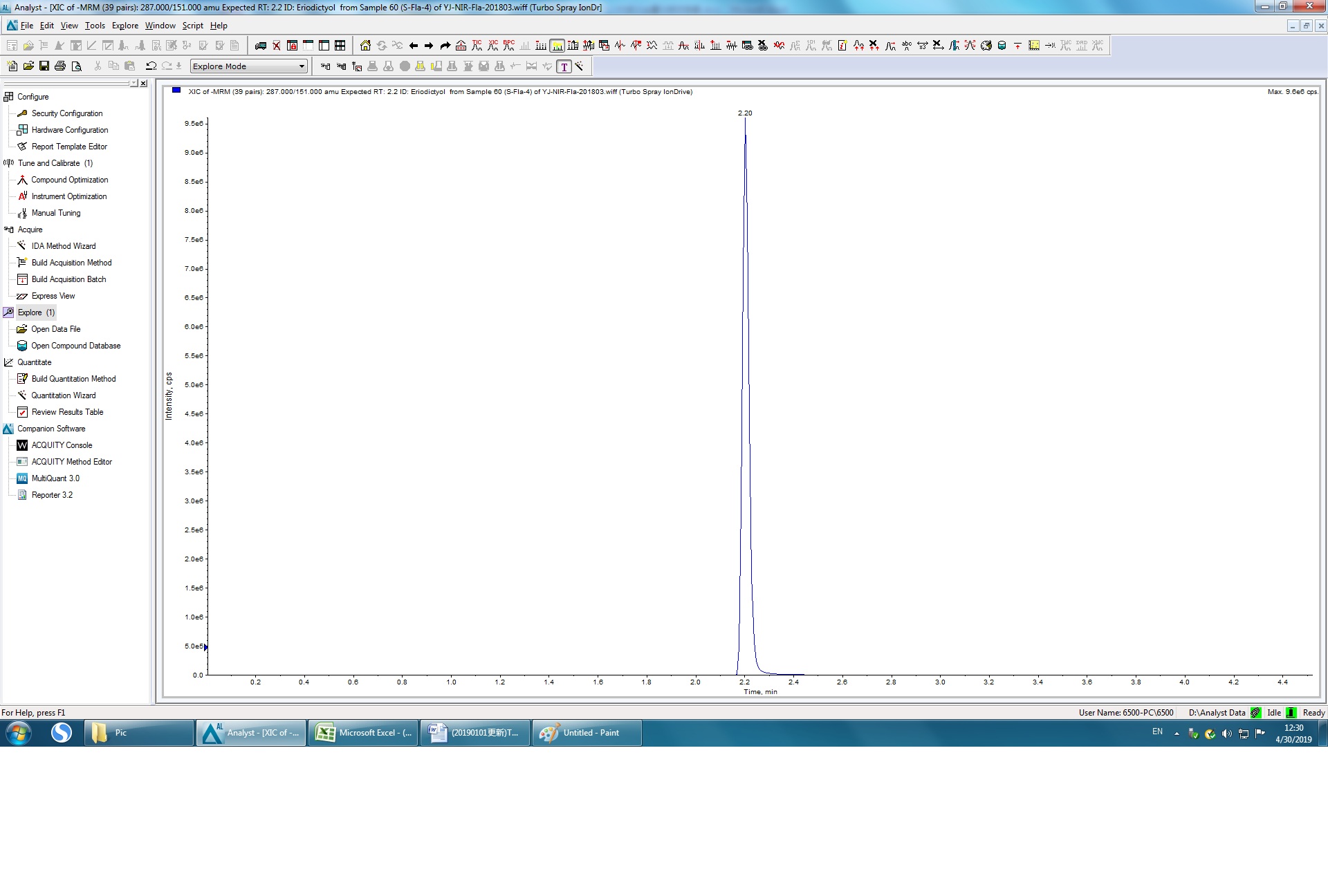

Supplement: Supplementary file 1 [file Data_Sheet_1.ZIP › Flow diagrams 926197/Eriodictyol.jpg]

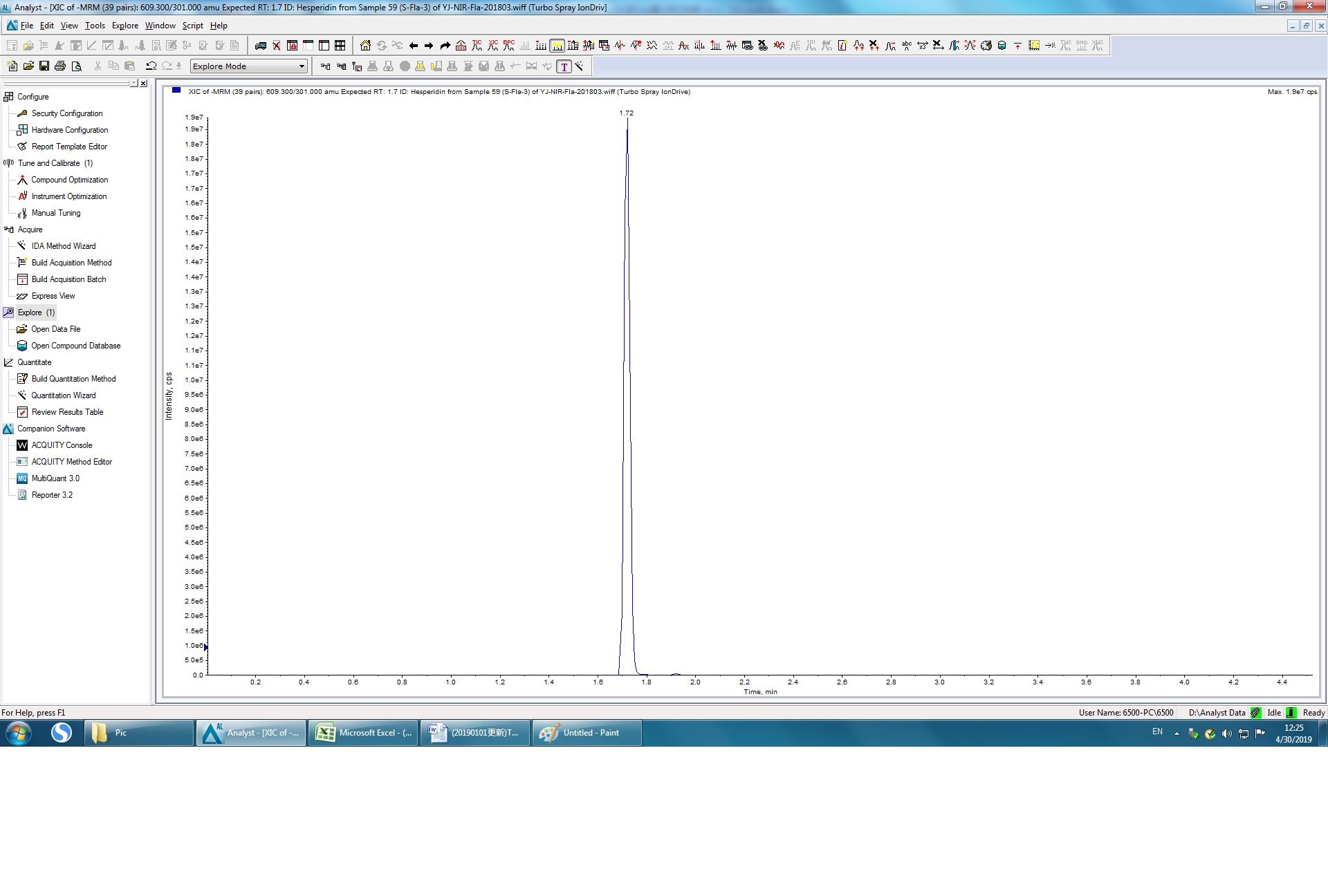

Supplement: Supplementary file 1 [file Data_Sheet_1.ZIP › Flow diagrams 926197/Hesperidin.jpg]

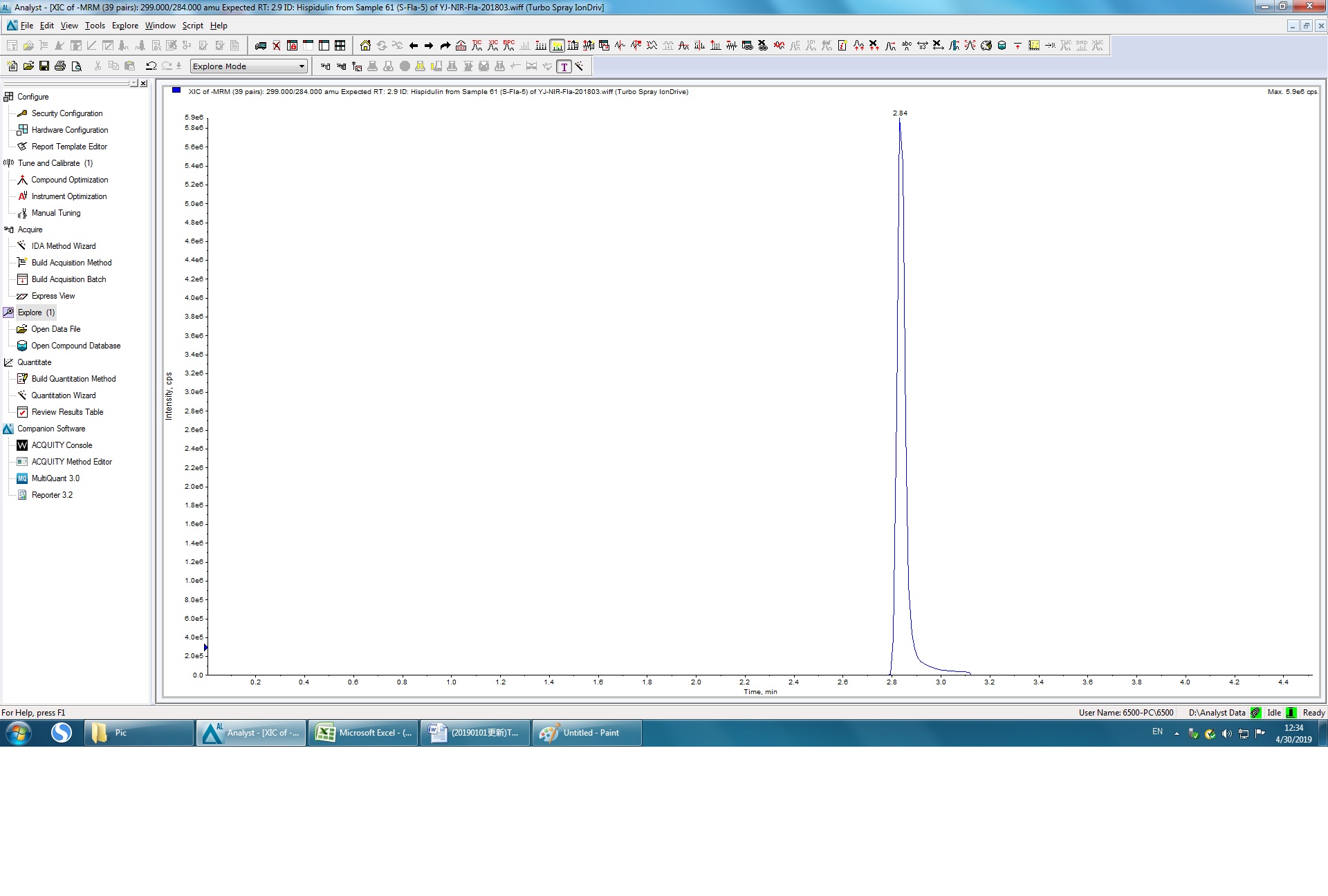

Supplement: Supplementary file 1 [file Data_Sheet_1.ZIP › Flow diagrams 926197/Hispidulin.jpg]

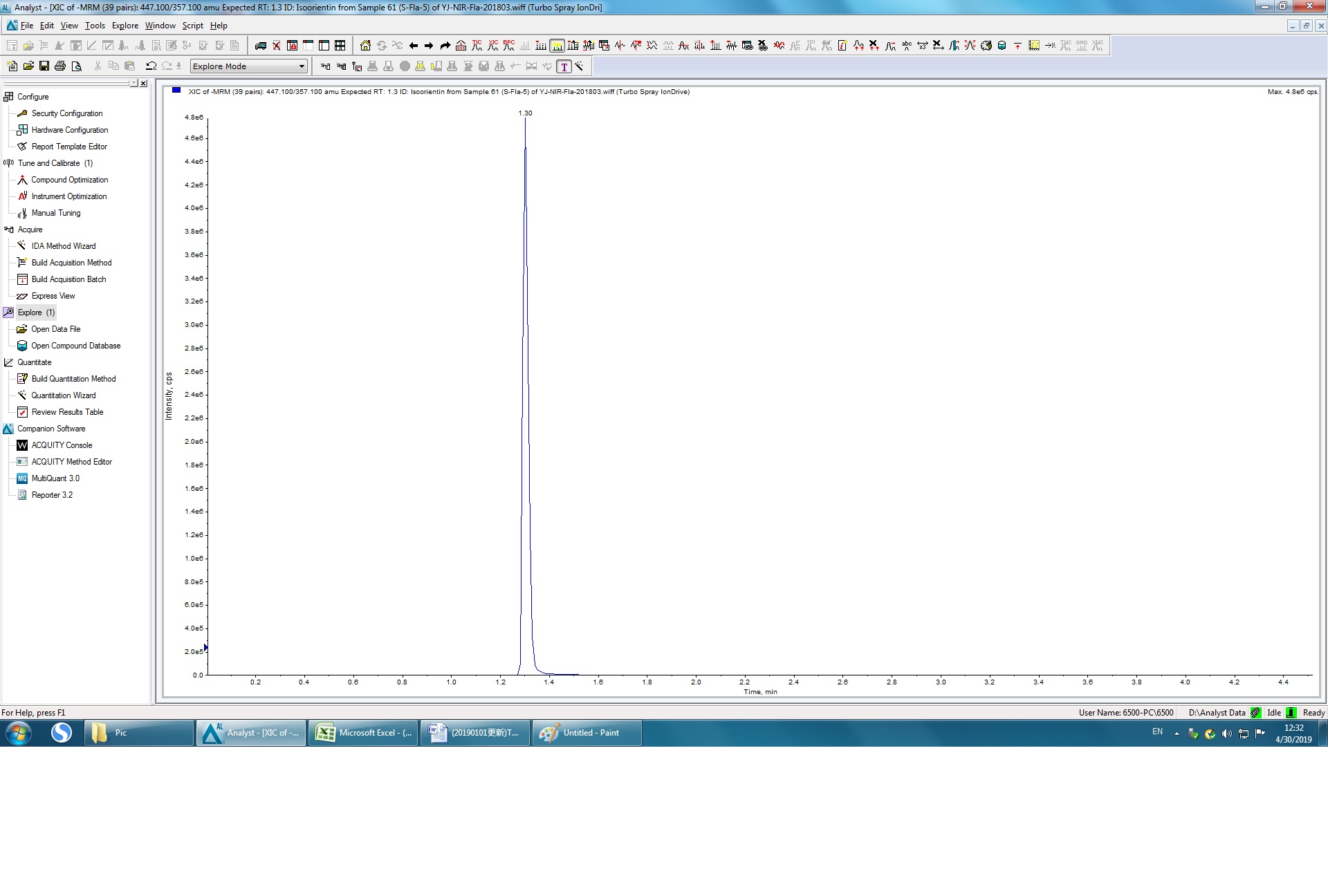

Supplement: Supplementary file 1 [file Data_Sheet_1.ZIP › Flow diagrams 926197/Isoorientin.jpg]

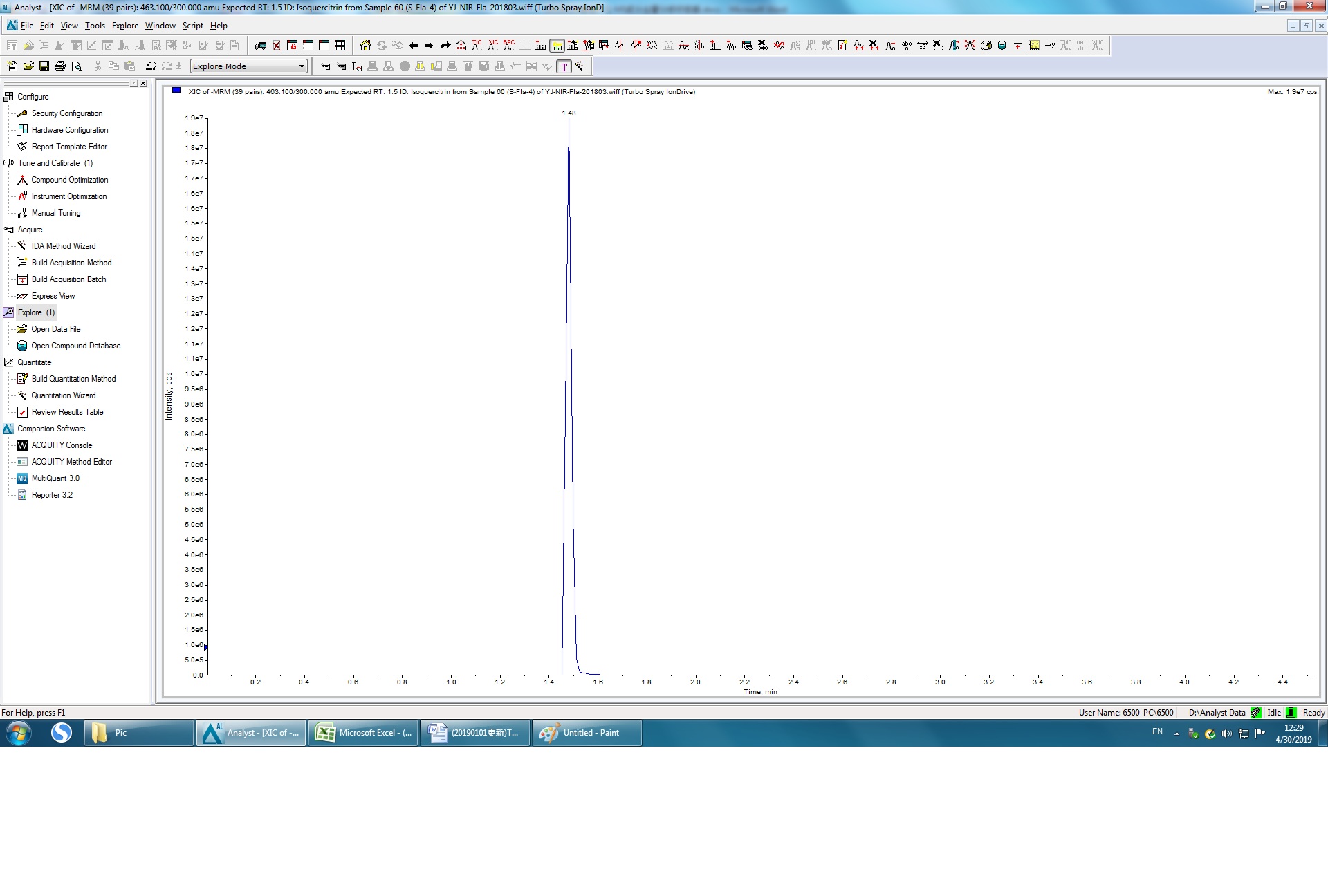

Supplement: Supplementary file 1 [file Data_Sheet_1.ZIP › Flow diagrams 926197/Isoquercitrin.jpg]

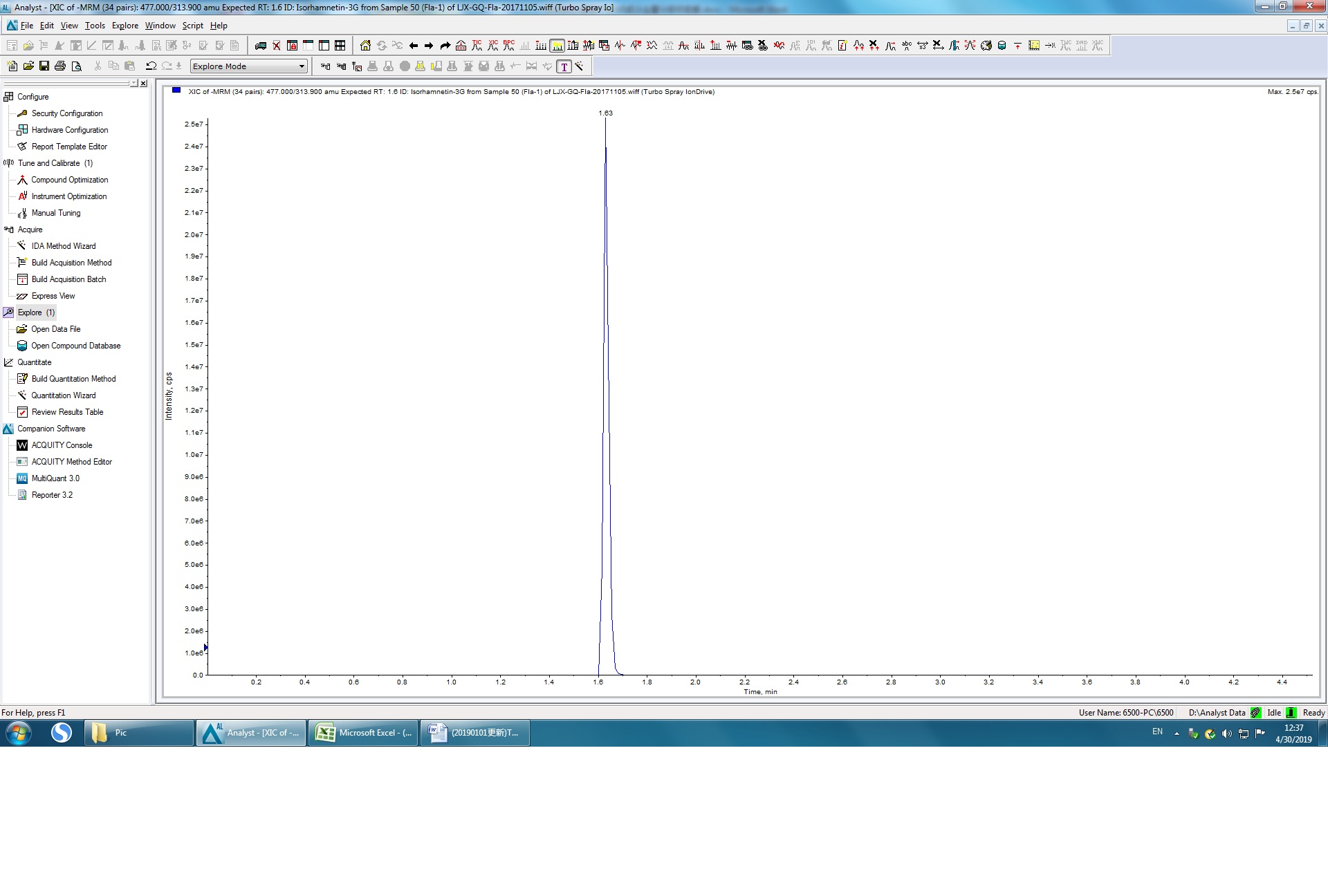

Supplement: Supplementary file 1 [file Data_Sheet_1.ZIP › Flow diagrams 926197/Isorhamnetin 3-O-glu.jpg]

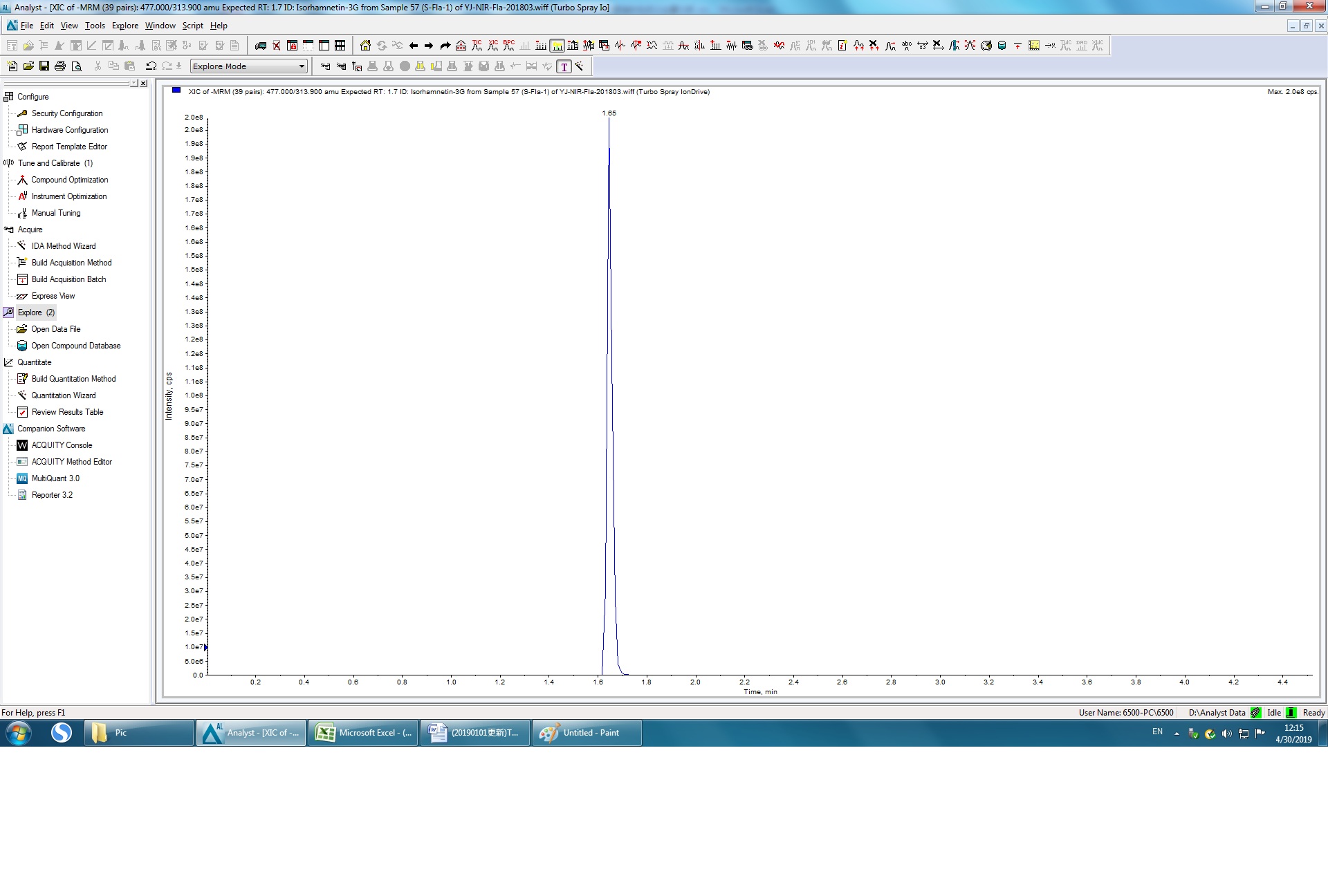

Supplement: Supplementary file 1 [file Data_Sheet_1.ZIP › Flow diagrams 926197/Isorhamnetin.jpg]

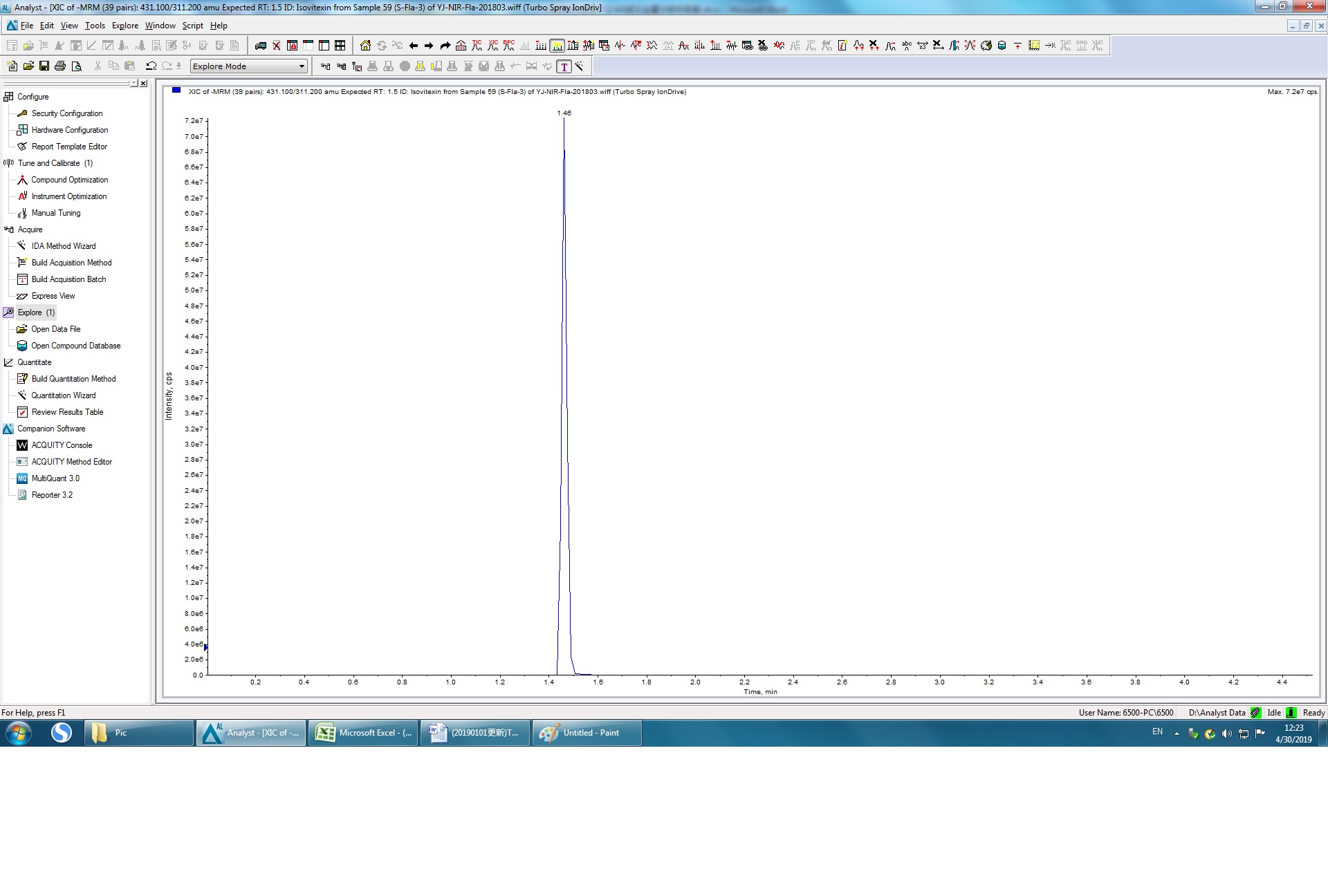

Supplement: Supplementary file 1 [file Data_Sheet_1.ZIP › Flow diagrams 926197/Isovitexin.jpg]

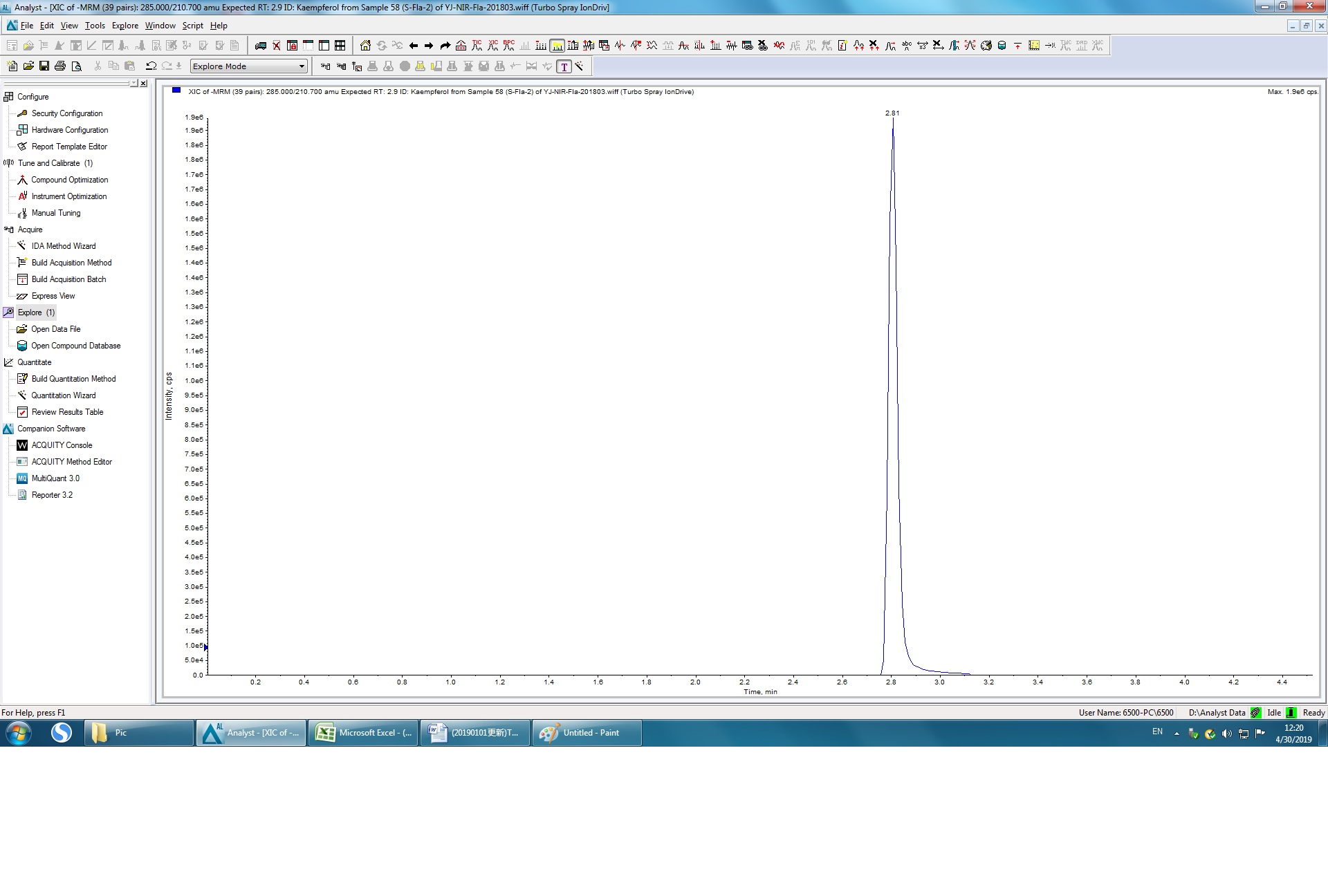

Supplement: Supplementary file 1 [file Data_Sheet_1.ZIP › Flow diagrams 926197/Kaempferol.jpg]

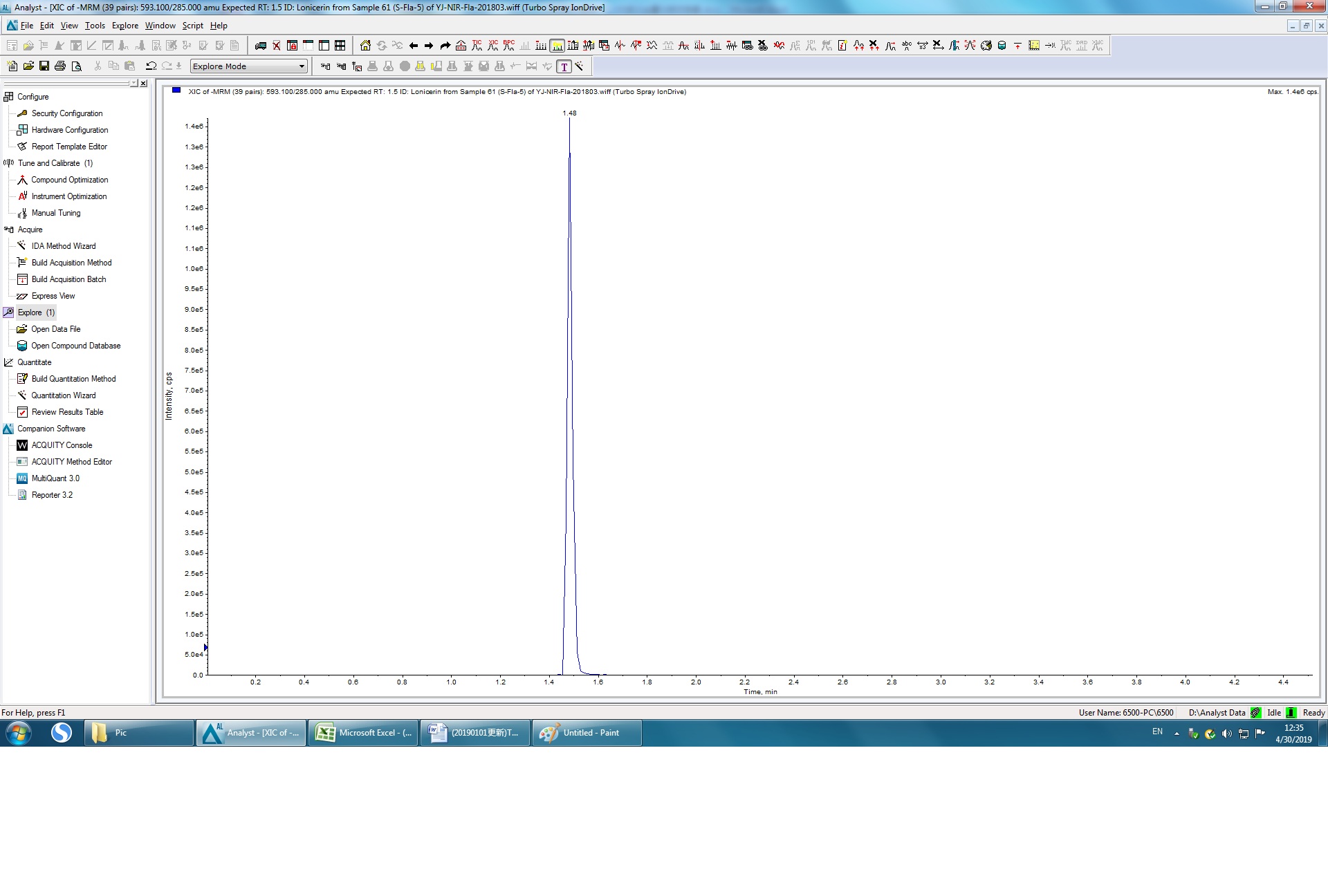

Supplement: Supplementary file 1 [file Data_Sheet_1.ZIP › Flow diagrams 926197/Lonicerin.jpg]

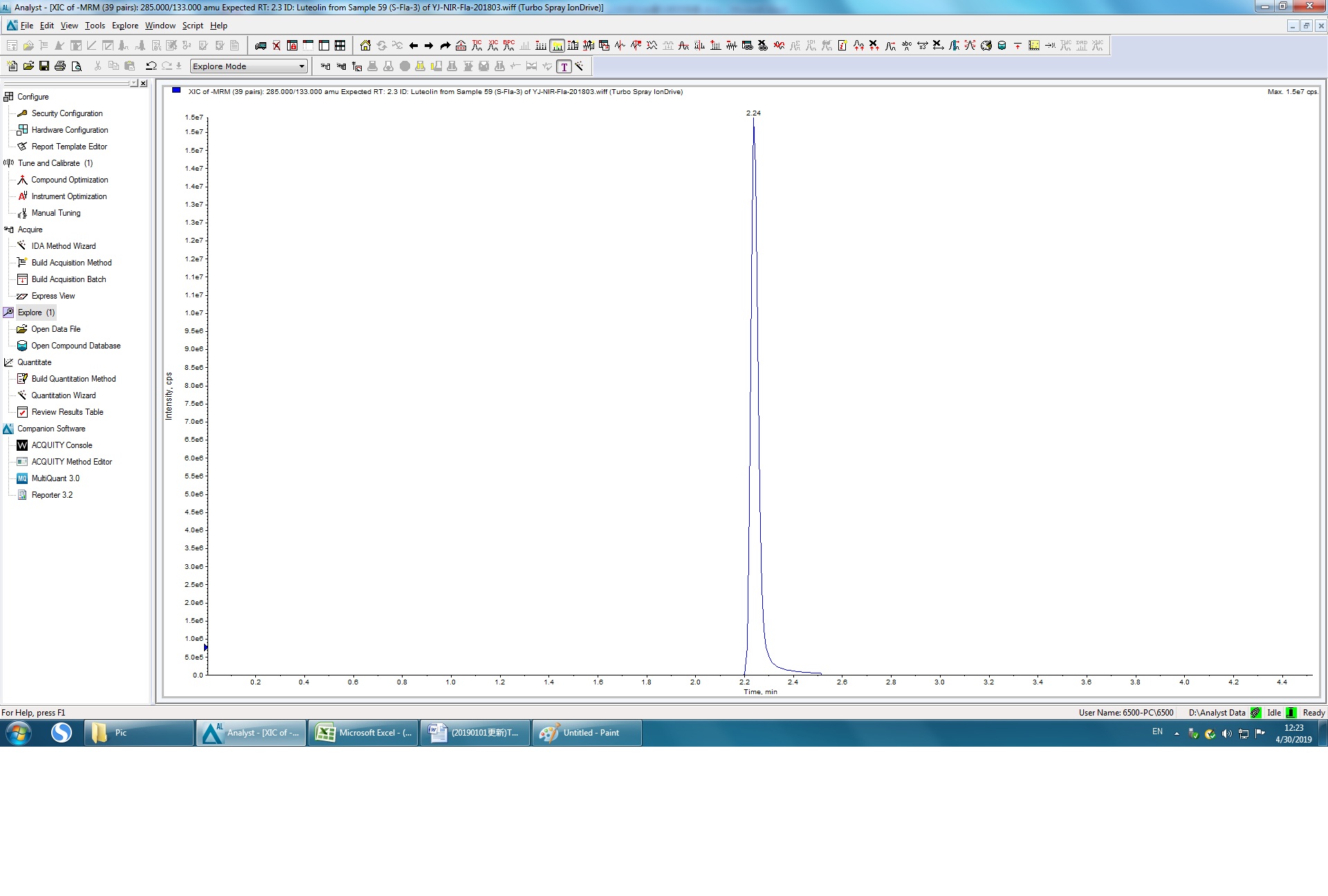

Supplement: Supplementary file 1 [file Data_Sheet_1.ZIP › Flow diagrams 926197/Luteolin.jpg]

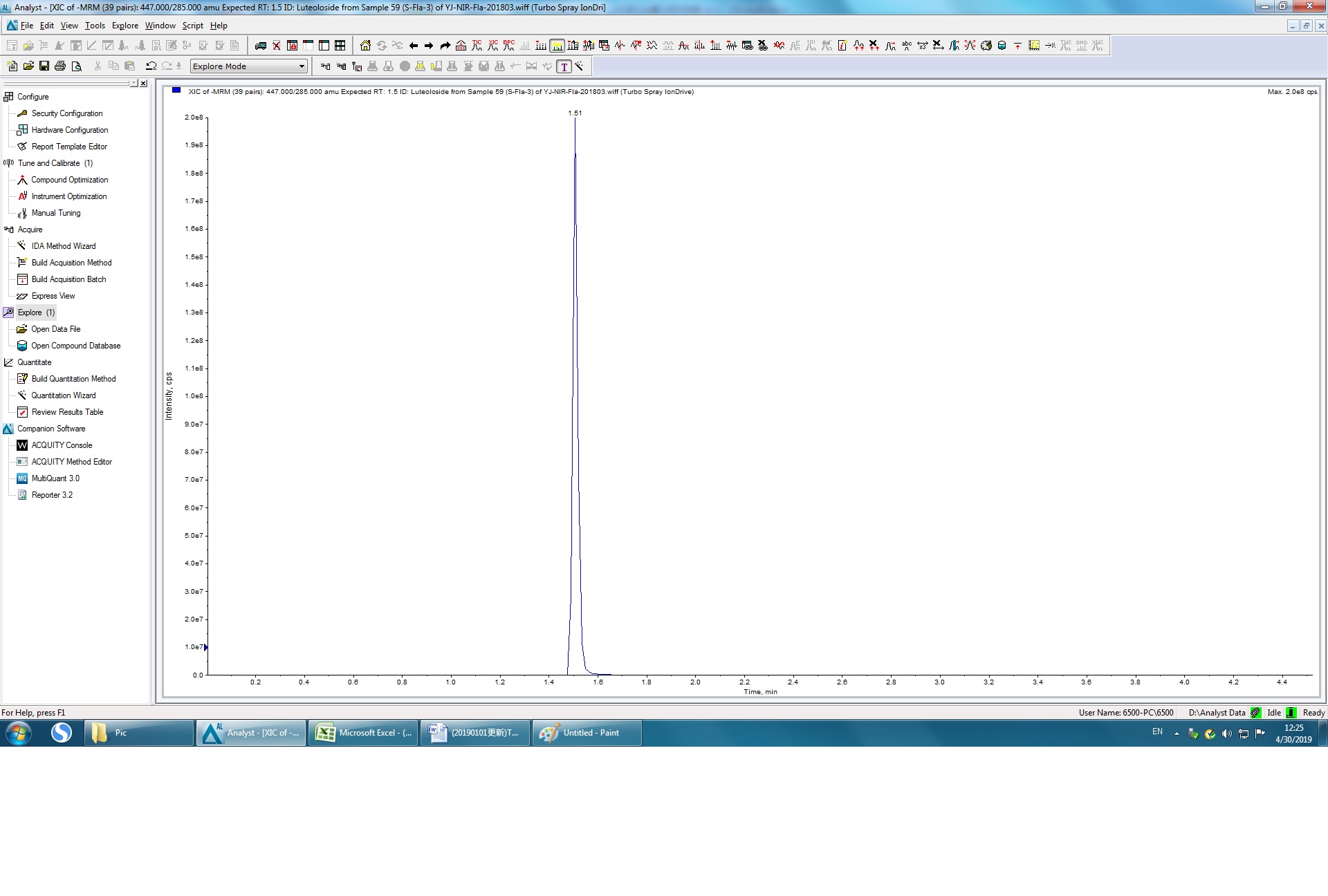

Supplement: Supplementary file 1 [file Data_Sheet_1.ZIP › Flow diagrams 926197/Luteoloside.jpg]

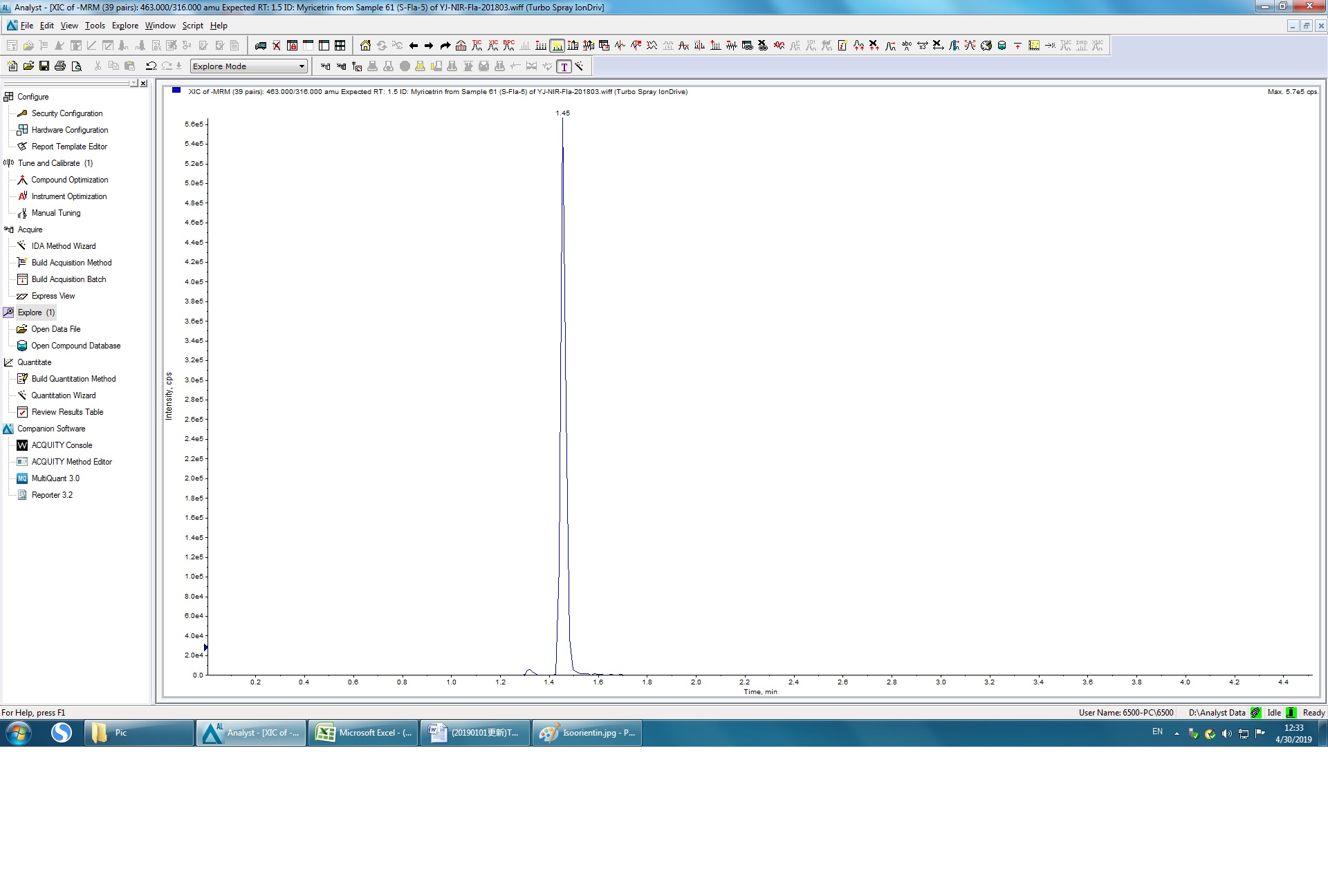

Supplement: Supplementary file 1 [file Data_Sheet_1.ZIP › Flow diagrams 926197/Myricetrin.jpg]

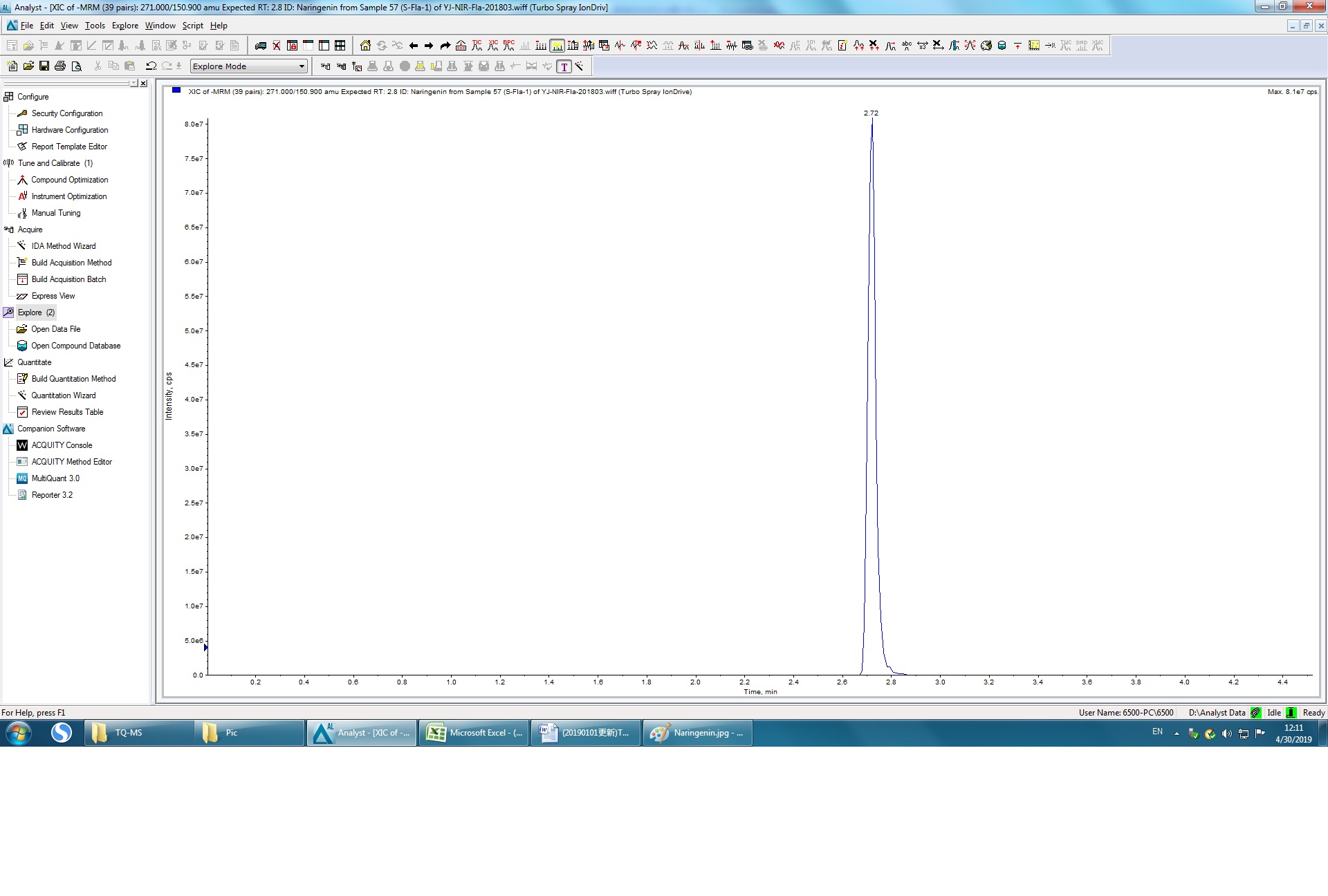

Supplement: Supplementary file 1 [file Data_Sheet_1.ZIP › Flow diagrams 926197/Naringenin.jpg]

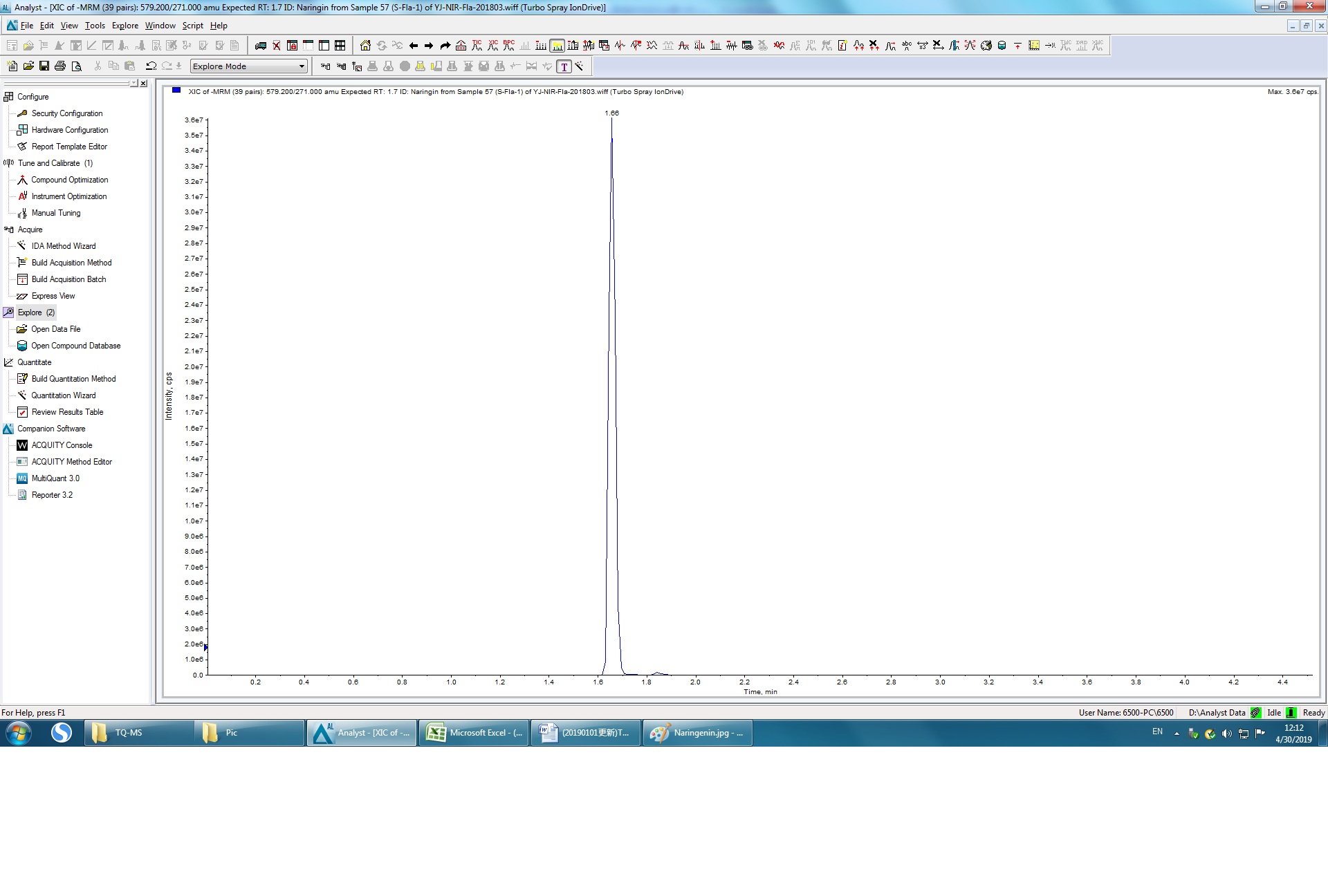

Supplement: Supplementary file 1 [file Data_Sheet_1.ZIP › Flow diagrams 926197/Naringin.jpg]

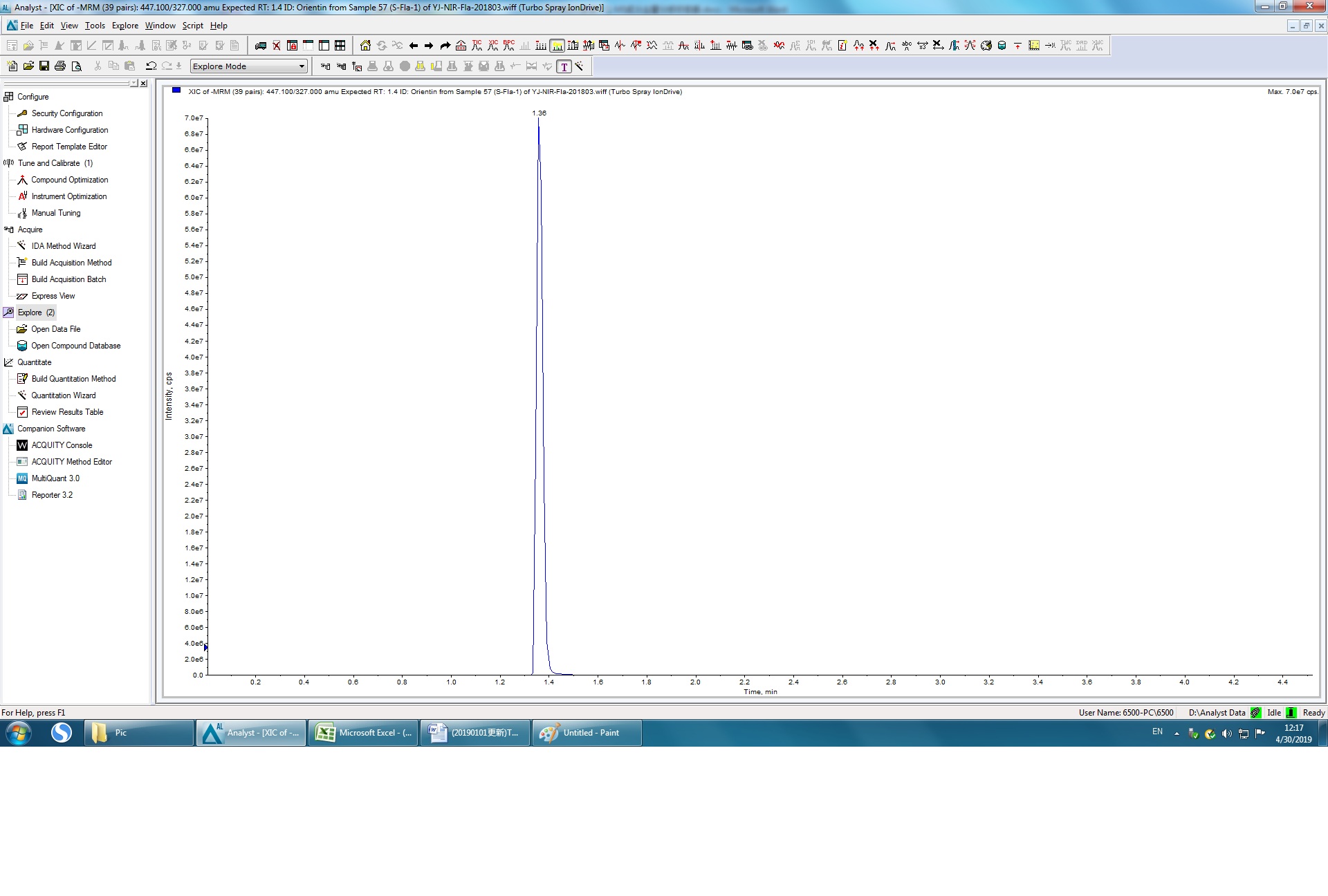

Supplement: Supplementary file 1 [file Data_Sheet_1.ZIP › Flow diagrams 926197/Orientin.jpg]

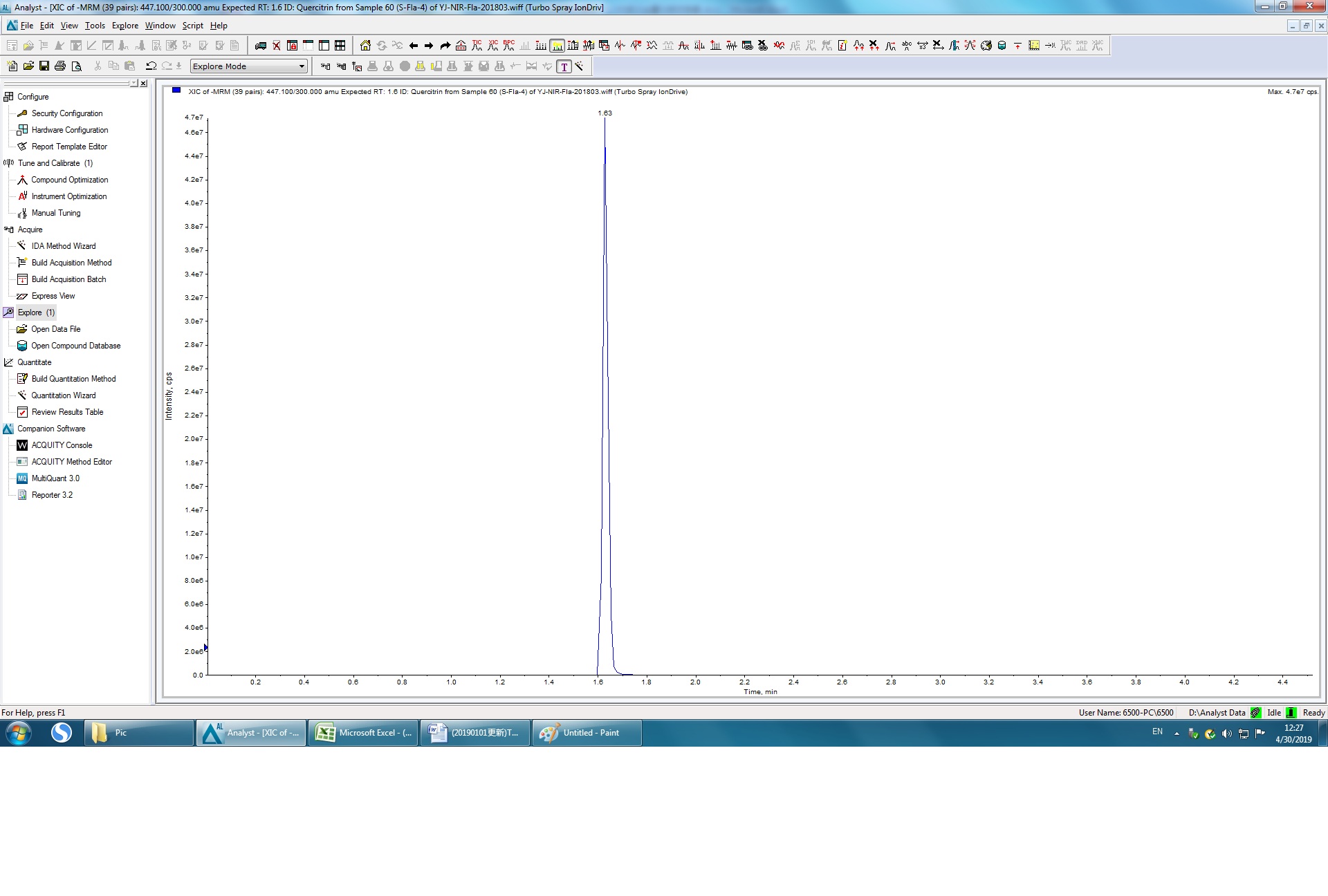

Supplement: Supplementary file 1 [file Data_Sheet_1.ZIP › Flow diagrams 926197/Quercitrin.jpg]

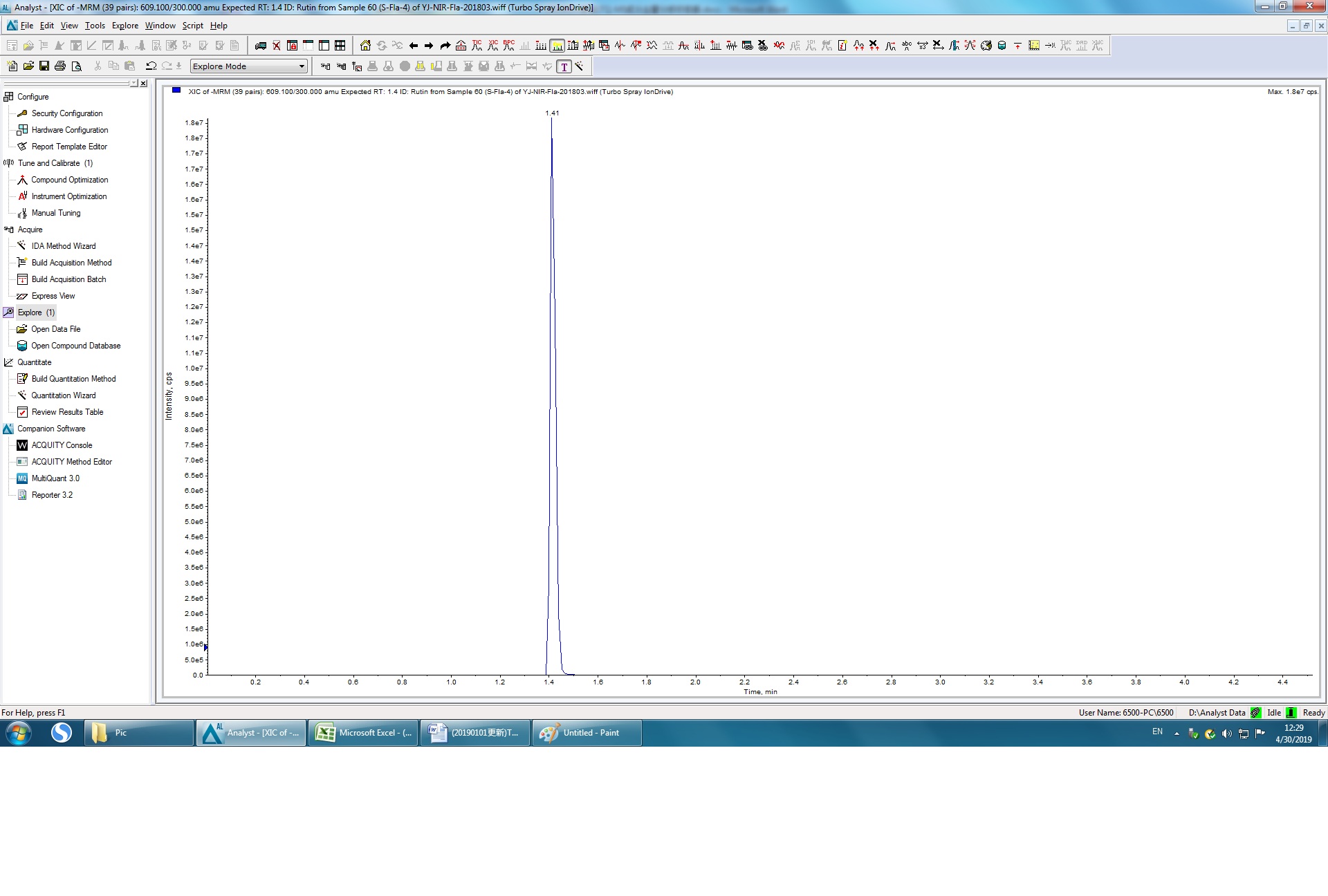

Supplement: Supplementary file 1 [file Data_Sheet_1.ZIP › Flow diagrams 926197/Rutin.jpg]

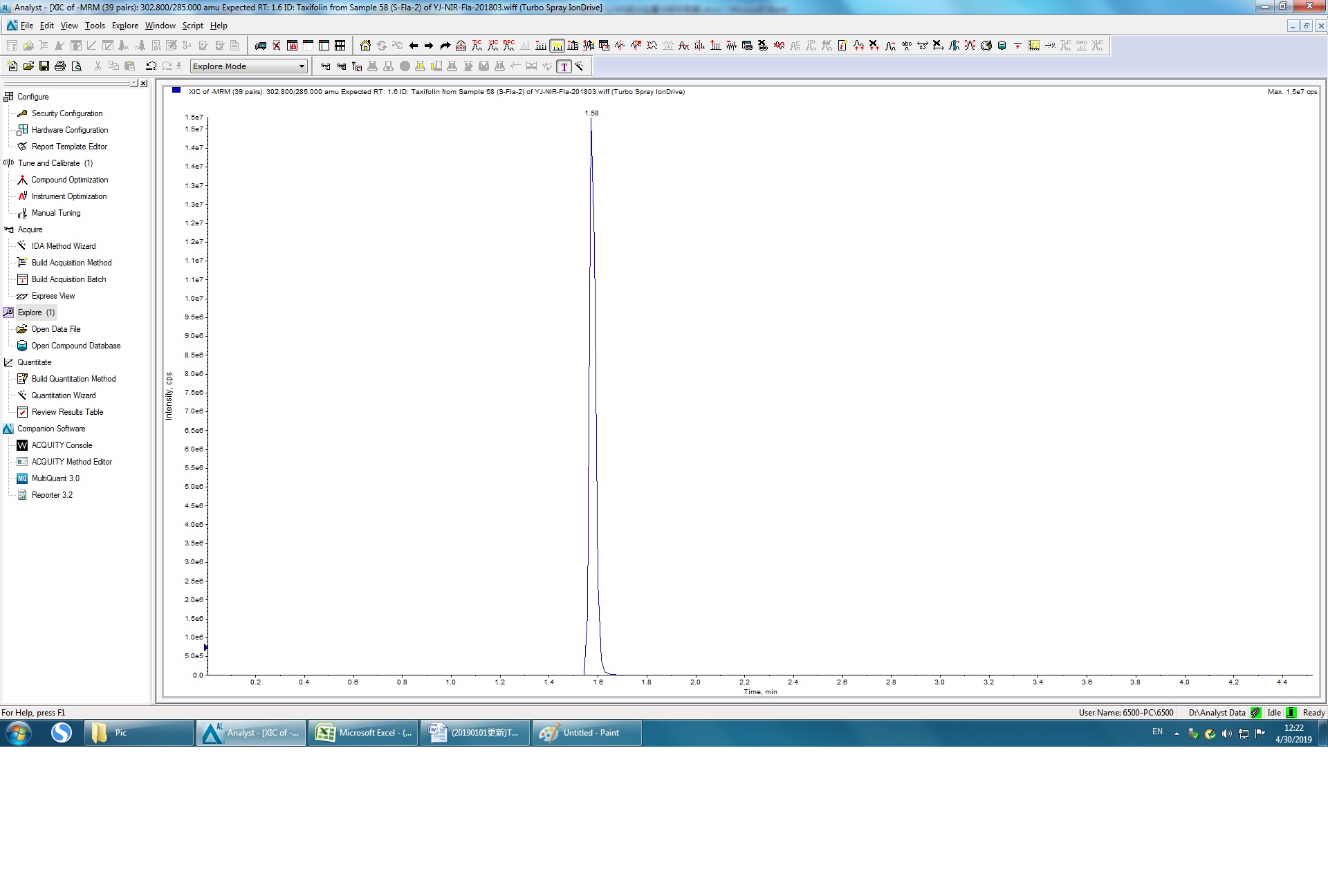

Supplement: Supplementary file 1 [file Data_Sheet_1.ZIP › Flow diagrams 926197/Taxifolin.jpg]
