# Supplementary material for: Flavonoids metabolism and physiological response to ultraviolet treatments in Tetrastigma hemsleyanum Diels et Gilg
Source: Front Plant Sci. 2022 Sep 15;13:926197. doi: 10.3389/fpls.2022.926197 (PMC9520580; doi:10.3389/fpls.2022.926197)
Supplement: Supplementary file 2 [file Data_Sheet_2.docx]

Supplementary Material

# Supplementary Figures and Tables

## Supplementary Figures


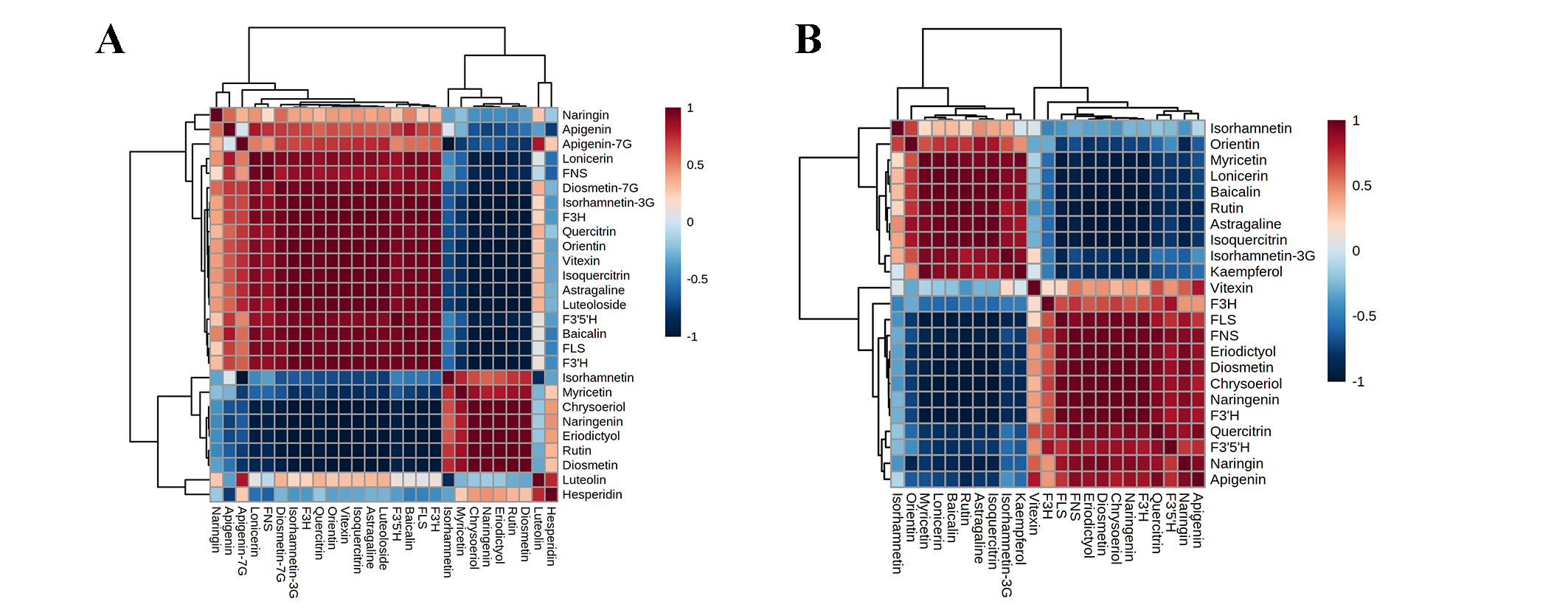


Supplementary Fig 1 Correlation analysis of flavonoid monomers contents and flavonoid synthesis related enzymes activities in leaves(A) and root tubers(B) of *T. hemsleyanum*

## Supplementary Tables

**Supplementary Table 1** Optimized LC-MS /MS parameters of flavonoids

| **Name** | **Rt (min)** | **MRM** | | | |
| --- | --- | --- | --- | --- | --- |
|  |  | **MRM ion pairs (m/z)** | **Declustering potential (DP/V)** | **Collision energy (CE/eV)** | **Injection voltage (CXP/V)** |
| Naringenin | 2.72 | 271.0/150.9 | -85 | -25 | -17 |
| Naringin | 1.67 | 579.2/271.0 | -217 | -42 | -8 |
| Isorhamnetin | 3.00 | 315.0/299.8 | -25 | -29 | -22 |
| Isorhamnetin 3-*O*-glucoside | 1.56 | 477.0/313.9 | -13.5 | -37 | -21 |
| Afzelin | 1.84 | 431.1/285.0 | -122 | -27 | -15 |
| Orientin | 1.36 | 447.1/327.0 | -173 | -30 | -27 |
| Isoorientin | 1.30 | 447.1/357.1 | -166 | -31 | -7 |
| Apigenin | 2.76 | 269.1/117.0 | -171 | -43 | -19 |
| Apigenin 7-*O*-glucoside | 1.69 | 431.1/268.0 | -266 | -47 | -40 |
| Astragaline | 1.61 | 447.1/254.8 | -170 | -46 | -23 |
| Taxifolin | 1.58 | 302.8/285.0 | -152 | -19 | -9 |
| Luteolin | 2.24 | 285.0/133.0 | -144 | -40 | -15 |
| Luteoloside | 1.51 | 447.0/285.0 | -146 | -34 | -25 |
| Isovitexin | 1.46 | 431.1/311.2 | -11 | -37 | -27 |
| Hesperidin | 1.72 | 609.3/301.0 | -197 | -31 | -30 |
| Calycosin 7-*O*- glucoside | 1.48 | 445.1/283.0 | -29 | -23 | -40 |
| Quercitrin | 1.63 | 447.1/300.0 | -97 | -34 | -36 |
| Isoquercitrin | 1.48 | 463.1/300.0 | -191 | -36 | -22 |
| Rutin | 1.41 | 609.1/300.0 | -245 | -48 | -38 |
| Eriodictyol | 2.20 | 287.0/151.0 | -71 | -21 | -12 |
| Hispidulin | 2.84 | 299.0/284.0 | -4 | -28 | -25 |
| Lonicerin | 1.48 | 593.1/285.0 | -298 | -55 | -32 |
| Avicularin | 1.57 | 433.0/301.0 | -15 | -27 | -32 |

**Supplementary Table 2** Results of linear relationship

| **Name** | **Regression equation** | **R value** | **Linear range (μg/mL)** |
| --- | --- | --- | --- |
| Naringenin | Y = 12452147.9 X + 74631.9 | 0.9992 | 0.001~0.050 |
| Naringin | Y = 3972039.5 X + 64183.6 | 0.9976 | 0.010~0.500 |
| Isorhamnetin | Y = 1476424.2 X + 2111521.6 | 0.9986 | 0.001 ~ 0.050 |
| Isorhamnetin 3-O-glucoside | Y = 8988517.2 X + 5992231.1 | 0.9982 | 0.020 ~ 1.000 |
| Afzelin | Y = 9658807.6 X + 92259.2 | 0.9993 | 0.001 ~ 0.050 |
| Orientin | Y = 9071337.1 X + 33093.6 | 0.9996 | 1.00 ~ 100.0 |
| Isoorientin | Y = 4845698.3 X + 4222807.8 | 0.9987 | 1.00 ~ 100.0 |
| Apigenin | Y = 2885631.5 X + 238287.7 | 0.9989 | 0.001 ~ 0.050 |
| Apigenin 7-O-glucoside | Y = 1806515.0 X + 16658.0 | 0.9996 | 0.010 ~ 0.500 |
| Astragaline | Y = 2210508.5 X + 2507321.4 | 0.9986 | 0.010 ~ 0.500 |
| Taxifolin | Y = 3575802.9 X + 284658.7 | 0.9992 | 0.001 ~ 0.050 |
| Luteolin | Y = 9109434.3 X + 35359.9 | 0.9998 | 0.020 ~ 1.000 |
| Luteoloside | Y = 3168802.6 X + 166068.3 | 0.9994 | 0.10 ~ 5.00 |
| Isovitexin | Y = 8542235.9 X + 18454664.6 | 0.9987 | 20.0 ~ 2000.0 |
| Hesperidin | Y = 12553808.9 X + 77366.1 | 0.9986 | 0.020 ~ 1.000 |
| Calycosin 7-O- glucoside | Y = 22042.7 X + 2463.8 | 0.9986 | 0.020 ~ 1.000 |
| Quercitrin | Y = 12206204.2 X + 1425428.8 | 0.9992 | 0.001 ~ 0.050 |
| Isoquercitrin | Y = 3356305.1 X + 593847.8 | 0.9989 | 0.020 ~ 1.000 |
| Rutin | Y = 4950860.8 X + 231551.2 | 0.9994 | 0.010 ~ 0.500 |
| Eriodictyol | Y = 691932.1 X + 84659.5 | 0.9991 | 0.010 ~ 0.500 |
| Hispidulin | Y = 207676.0 X + 105248.9 | 0.9993 | 0.020 ~ 1.000 |

**Supplementary Table 3** **Effect of UV treatment on flavonoid monomers of leaves in *T. hemsleyanum* (ug/g)**

|  | Isovitexin | | Orientin | | Lonicerin | | Luteoloside | | Luteolin | | Isoquercitrin | |  |
| --- | --- | --- | --- | --- | --- | --- | --- | --- | --- | --- | --- | --- | --- |
| CK | | 6282.4±528.601ij | | 187.184±10.318fg | | 29.582±2.272b | | 3.518±0.491i | | 3.891±0.879c | | 4.085±0.418de | |
| UV-A (15min) | | 10378.6±486.575f | | 296.153±33.759b | | 22.58±2.637d | | 23.78±2.85a | | 2.576±0.45e | | 8.205±1.053b | |
| UV-A (30min) | | 6097.15±69.573ij | | 213.861±7.663de | | 15.477±0.321e | | 13.306±1.001bc | | 1.216±0.057f | | 4.005±0.171de | |
| UV-A (1h) | | 6119.5±84.512ij | | 102.737±2.428j | | 7.314±0.368g | | 1.179±0.074jk | | 0.836±0.076fg | | 0.06±0.024h | |
| UV-A (2h) | | 3571.25±123.522l | | 52.077±1.097k | | 2.79±0.054h | | 2.801±0.018ij | | 0.033±0.001i | | 0.24±0.024gh | |
| UV-A (3h) | | 7959.25±120.987h | | 162.121±0.815gh | | 4.63±0.136gh | | 5.821±0.204h | | 0.279±0.04gh | | 0.505±0.031gh | |
| UV-A (5h) | | 5452.05±0k | | 136.1±0ij | | 5.859±0gh | | 0.809±0.005k | | 0.731±0.002fg | | 0.765±0gh | |
| UV-B (15min) | | 15234.5±229.976b | | 244.259±34.766cd | | 16.164±0.376e | | 12.356±0.382cd | | 2.559±0.026e | | 4.54±0.092d | |
| UV-B (30min) | | 14142.7±305.758c | | 266.113±4.741bc | | 12.265±0.756f | | 3.472±0.243i | | 0.151±0.014hi | | 3.31±0.148ef | |
| UV-B (1h) | | 26329.2±500.672a | | 461.123±16.835a | | 23.021±1.285d | | 12.512±0.503c | | 2.81±0.279de | | 10.835±0.614a | |
| UV-B (2h) | | 5794.55±166.259jk | | 144.772±2.329hi | | 3.968±0.01h | | 7.304±0.145gh | | 0.346±0.007gh | | 0.97±0.019g | |
| UV-B (3h) | | 9337.4±170.977g | | 201.514±3.175ef | | 30.45±2.167ab | | 15.016±0.267b | | 2.53±0.15e | | 3.955±0.198de | |
| UV-B (5h) | | 6590.6±183.558i | | 162.413±4.635gh | | 3.429±0.155h | | 1.392±0.073jk | | 0.251±0.013gh | | 2.51±0.086f | |
| UV-C (15min) | | 8743.35±367.919g | | 213.249±37.563de | | 23.43±1.219cd | | 10.056±0.602ef | | 3.39±0.135cd | | 8.69±0.363b | |
| UV-C (30min) | | 6282.4±111.285ij | | 194.349±2.807ef | | 31.086±1.465ab | | 13.435±0.516bc | | 5.301±0.153a | | 2.625±0.042f | |
| UV-C (1h) | | 6517.95±475.323ij | | 184.814±7.831fg | | 26.27±0.488c | | 8.403±0.312fg | | 4.675±0.224ab | | 4.69±0.263d | |
| UV-C (2h) | | 12849.15±64.04de | | 229.184±28.869cd | | 31.16±1.11ab | | 10.147±0.43ef | | 4.638±0.245b | | 5.825±0.192c | |
| UV-C (3h) | | 13077.5±355.109d | | 207.137±4.327de | | 33.483±1.821a | | 8.999±0.29ef | | 4.993±0.072ab | | 3.505±0.067e | |
| UV-C (5h) | | 12248.7±162.301e | | 209.837±0.868de | | 20.814±0.556d | | 10.478±0.223de | | 2.813±0.121de | | 2.57±0.106f | |

**Supplementary Table 3** **Effect of UV treatment on flavonoid monomers of leaves in *T. hemsleyanum* (ug/g) (continued)**

|  | Rutin | Hesperidin | Naringin | Isorhamnetin | Isorhamnetin 3-O-glucoside | Astragaline |
| --- | --- | --- | --- | --- | --- | --- |
| CK | 11.373±1.018a | 3.107±0.482bc | 1.51±0.131c | 0.449±0.08a | 0.685±0.018e | 0.323±0.031e |
| UV-A (15min) | 1.235±0.111ef | 2.103±0.165ef | 0.778±0.091f | 0.295±0.026b | 0.839±0.039cd | 1.599±0.08a |
| UV-A (30min) | 1.632±0.108e | 1.882±0.116f | 0.509±0.023g | 0.232±0.004cd | 0.57±0.003hi | 0.232±0.006gh |
| UV-A (1h) | 0.694±0.027fg | 0.952±0.056gh | 0.267±0.02hi | 0.225±0.003cd | 0.551±0.002i | 0.24±0.011fg |
| UV-A (2h) | 0.054±0.002gh | 0.111±0.006j | 0.158±0.004i | 0.216±0.004ef | 0.536±0.002i | 0.188±0.007i |
| UV-A (3h) | 0.076±0.003gh | 0.324±0.02ij | 0.189±0.001i | 0.21±0.001fg | 0.559±0.003hi | 0.256±0.011fg |
| UV-A (5h) | 0.034±0.001h | 0.395±0.004ij | 0.387±0.002gh | 0.198±0g | 0.593±0gh | 0.203±0hi |
| UV-B (15min) | 1.444±0.051e | 1.268±0.026g | 0.798±0.001f | 0.272±0.001bc | 0.682±0.002e | 0.634±0.019c |
| UV-B (30min) | 0.533±0.056gh | 1.107±0.066g | 0.41±0.021g | 0.203±0.004fg | 0.617±0.001fg | 0.333±0.004e |
| UV-B (1h) | 0.122±0.01gh | 2.008±0.126f | 1.037±0.057e | 0.212±0.009fg | 1.076±0.031a | 0.657±0.054bc |
| UV-B (2h) | 0.104±0.005gh | 0.206±0.024j | 0.213±0.003i | 0.197±0.001g | 0.561±0.001hi | 0.325±0.003e |
| UV-B (3h) | 4.123±0.211c | 2.757±0.101cd | 2.305±0.09a | 0.198±0.004g | 0.607±0.002g | 0.296±0.008ef |
| UV-B (5h) | 0.47±0.004gh | 0.61±0.048hi | 0.402±0.006g | 0.216±0.002ef | 0.588±0.001gh | 0.332±0.004e |
| UV-C (15min) | 0.359±0.04gh | 2.409±0.103de | 0.774±0.032f | 0.248±0.001bc | 0.917±0.013b | 0.304±0.015ef |
| UV-C (30min) | 3.357±0.098d | 4.323±0.069a | 1.576±0.035c | 0.265±0.016bc | 0.614±0.001fg | 0.355±0.017e |
| UV-C (1h) | 1.241±0.169ef | 3.466±0.091b | 1.375±0.053d | 0.434±0.018a | 0.804±0.023d | 0.593±0.019c |
| UV-C (2h) | 4.571±0.278c | 2.019±0.108f | 2.226±0.075a | 0.254±0.006bc | 0.863±0.016c | 0.716±0.023b |
| UV-C (3h) | 5.562±0.153b | 2.823±0.293c | 1.717±0.014b | 0.27±0.009bc | 0.85±0.013c | 0.486±0.001d |
| UV-C (5h) | 1.597±0.045e | 1.728±0.045f | 0.963±0.003e | 0.22±0.001de | 0.649±0.007ef | 0.441±0.006d |

**Supplementary Table 3** **Effect of UV treatment on flavonoid monomers of leaves in *T. hemsleyanum* (ug/g) (continued)**

|  | Baicalin | Apigenin 7-O-glucoside | Quercitrin | Chrysoeriol | Apigenin |
| --- | --- | --- | --- | --- | --- |
| CK | 0.104±0.028d | 0.031±0.02kl | 0.031±0.003c | 0.087±0.013de | 0.042±0.01bc |
| UV-A (15min) | 0.154±0.044d | 0.279±0.035b | 0.027±0.001c | 0.008±0.001hi | 0.044±0.01ab |
| UV-A (30min) | 0.104±0.008d | 0.169±0.014ef | 0.033±0.008c | 0.008±0.001hihi | 0.031±0.003fg |
| UV-A (1h) | 0.067±0.031d | 0.032±0.009kl | 0.024±0c | 0.007±0.001 | 0.024±0.002hi |
| UV-A (2h) | 0.051±0.002d | 0.073±0.009ij | 0.023±0.001c | 0.003±0j | 0.021±0.001ij |
| UV-A (3h) | 0.069±0.004d | 0.059±0.004jk | 0.17±0.015b | 0.005±0ij | 0.02±0.001jk |
| UV-A (5h) | 0.053±0.001d | 0±0l | 0.025±0.001c | 0.005±0.001ij | 0.019±0.001k |
| UV-B (15min) | 0.049±0.006d | 0.103±0.007hi | 0.027±0.001c | 0.08±0.002e | 0.052±0.001ab |
| UV-B (30min) | 0.109±0.004d | 0.028±0.01kl | 0.023±0c | 0.014±0gh | 0.03±0.003gh |
| UV-B (1h) | 0.47±0.072c | 0.052±0.004jk | 0.028±0.001c | 0.015±0.002gh | 0.042±0.009ab |
| UV-B (2h) | 0.155±0.001d | 0.073±0.002ij | 0.024±0.001c | 0.018±0.001g | 0.031±0.001fg |
| UV-B (3h) | 1.209±0.23b | 0.407±0.001a | 0.026±0.001c | 0.013±0.001gh | 0.05±0.006ab |
| UV-B (5h) | 1.659±0.084a | 0.011±0.005 | 0.026±0.001c | 0.01±0.001gh | 0.028±0.004gh |
| UV-C (15min) | 0.128±0.009d | 0.145±0.019fg | 0.025±0.002c | 0.042±0.002f | 0.032±0.004ef |
| UV-C (30min) | 0.127±0.019d | 0.211±0.028cd | 0.054±0.003c | 0.145±0.006a | 0.034±0.004de |
| UV-C (1h) | 0.146±0.001d | 0.134±0.01gh | 0.584±0.091a | 0.114±0.002b | 0.039±0.001cd |
| UV-C (2h) | 0.164±0.005d | 0.201±0.012cd | 0.031±0.001c | 0.081±0.004e | 0.051±0.001ab |
| UV-C (3h) | 0.117±0.018d | 0.22±0.009c | 0.027±0.002c | 0.094±0.003cd | 0.053±0.003a |
| UV-C (5h) | 0.591±0.039c | 0.182±0.008de | 0.027±0.001c | 0.1±0.001c | 0.043±0.001ab |

**Supplementary Table 3** **Effect of UV treatment on flavonoid monomers of leaves in *T. hemsleyanum* (ug/g) (continued)**

|  | Naringenin | Eriodictyol | Diosmetin | Diosmetin-7-glucoside | Myricetin |
| --- | --- | --- | --- | --- | --- |
| CK | 0.053±0.01d | 0.173±0.039c | 0.08±0.002f | 0±0d | 0.185±0.01a |
| UV-A (15min) | 0.045±0.006ef | 0.017±0.002hi | 0.007±0.001kl | 0.035±0.005c | 0.071±0.014d |
| UV-A (30min) | 0.013±0.001h | 0.022±0.001gh | 0.007±0.001kl | 0.037±0.005bc | 0.015±0hi |
| UV-A (1h) | 0.016±0.001h | 0.011±0.001i | 0.007±0.001kl | 0±0d | 0±0i |
| UV-A (2h) | 0.019±0.001gh | 0.019±0.001hi | 0.003±0l | 0±0d | 0±0i |
| UV-A (3h) | 0.02±0.001gh | 0.037±0.001fg | 0.005±0l | 0±0d | 0.015±0hi |
| UV-A (5h) | 0.016±0.001h | 0.012±0.002hi | 0.004±0.001l | 0±0d | 0.015±0hi |
| UV-B (15min) | 0.052±0.001de | 0.103±0.001e | 0.087±0.002e | 0.068±0.033ab | 0.032±0.001ef |
| UV-B (30min) | 0.02±0.001gh | 0.047±0.002fg | 0.015±0.001ij | 0.017±0.001cd | 0.015±0hi |
| UV-B (1h) | 0.013±0.001h | 0.015±0.001hi | 0.016±0.003hi | 0.085±0.038a | 0.093±0.014c |
| UV-B (2h) | 0.082±0.002b | 0.165±0.005c | 0.02±0.001h | 0.014±0.001cd | 0.023±0.001gh |
| UV-B (3h) | 0.014±0.001h | 0.02±0.001hi | 0.015±0.001ij | 0.021±0.005cd | 0.024±0.002fg |
| UV-B (5h) | 0.04±0.001f | 0.082±0.004e | 0.011±0jk | 0±0d | 0.043±0.003e |
| UV-C (15min) | 0.026±0.001g | 0.054±0.003f | 0.043±0.002g | 0.039±0.003bc | 0.096±0.007c |
| UV-C (30min) | 0.056±0.003d | 0.199±0.012b | 0.148±0.001a | 0.037±0.006bc | 0.033±0.002ef |
| UV-C (1h) | 0.057±0.003d | 0.182±0.003bc | 0.123±0.001b | 0.025±0.003cd | 0.039±0.005ef |
| UV-C (2h) | 0.076±0.003bc | 0.085±0.004e | 0.085±0.004e | 0.039±0.009bc | 0.109±0.014bc |
| UV-C (3h) | 0.07±0.003c | 0.131±0.003d | 0.103±0.001d | 0.035±0.007c | 0.121±0.007b |
| UV-C (5h) | 0.176±0.001a | 0.283±0.011a | 0.108±0.003c | 0.044±0.011bc | 0.099±0.001c |

**Supplementary Table 4** **Effect of UV treatment on flavonoid monomers of root tubers in *T. hemsleyanum* (ug/g)**

|  | Naringenin | Naringin | Isorhamnetin | Isorhamnetin-3G | Chrysoeriol | Orientin |
| --- | --- | --- | --- | --- | --- | --- |
| CK | 0.086±0.003c | 0.008±0.001ij | 0.495±0.078hi | 0.671±0.006hi | 0.004±0b | 0.057±0.015i |
| UV-A (15min) | 0.034±0.004i | 0.011±0.001i | 0.721±0.044fg | 1.154±0.013f | 0±0e | 1.866±0.034a |
| UV-A (30min) | 0.024±0.001j | 0.01±0.001i | 0.275±0.01jk | 0.799±0.009gh | 0±0e | 0.156±0.003gh |
| UV-A (1h) | 0.047±0.001de | 0.063±0.004c | 0.248±0.008kl | 0.544±0i | 0±0e | 0.163±0.014fg |
| UV-A (2h) | 0.118±0.001b | 0.124±0.001a | 0.22±0.007l | 0.596±0.004i | 0±0e | 0.943±0.026cd |
| UV-A (3h) | 0.043±0.001ef | 0.034±0.003ef | 0.457±0.013hi | 3.113±0.111b | 0±0e | 0.29±0.025fg |
| UV-A (5h) | 0.086±0.001c | 0±0j | 1.669±0.017d | 2.07±0.094c | 0±0e | 0.24±0.008fg |
| UV-B (15min) | 0.046±0.004ef | 0.036±0.004de | 0.427±0.044hi | 0.998±0.048fg | 0.002±0.001cd | 0.53±0.196e |
| UV-B (30min) | 0.043±0.003ef | 0.016±0.001hi | 2.048±0.215c | 3.74±0.289a | 0.002±0d | 0.077±0.003i |
| UV-B (1h) | 0.053±0.001d | 0.01±0.001i | 1.059±0.093e | 1.699±0.083d | 0.001±0d | 0.174±0.016fg |
| UV-B (2h) | 0.159±0.002a | 0.04±0.003de | 0.579±0.009gh | 1.703±0.044d | 0.002±0d | 0.829±0.01d |
| UV-B (3h) | 0.042±0.001fg | 0.098±0.008b | 0.219±0.001l | 0.551±0.001i | 0.002±0c | 0.318±0.016f |
| UV-B (5h) | 0.042±0.004fg | 0.034±0.011ef | 0.809±0.072f | 1.432±0.033e | 0±0e | 1.388±0.171b |
| UV-C (15min) | 0.017±0.001k | 0.016±0.002hi | 0.233±0.001l | 0.555±0.001i | 0±0e | 0.126±0.005hi |
| UV-C (30min) | 0.037±0hi | 0.023±0.001gh | 6.149±0.066a | 2.14±0.006c | 0±0e | 0.092±0.032hi |
| UV-C (1h) | 0.022±0.001jk | 0.026±0.002fg | 0.563±0.008gh | 0.709±0.006hi | 0±0e | 0.489±0.026e |
| UV-C (2h) | 0.048±0.006de | 0.044±0.004d | 2.832±0.154b | 1.041±0.013f | 0.005±0a | 1.022±0.011c |
| UV-C (3h) | 0.008±0l | 0.013±0.002hi | 0.371±0.015ij | 0.668±0.011hi | 0±0e | 0.017±0.001i |
| UV-C (5h) | 0.04±0.002gh | 0.014±0.003hi | 1.794±0.073d | 1.148±0.026f | 0.002±0cd | 0.026±0.008i |

**Supplementary Table 4** **Effect of UV treatment on flavonoid monomers of root tubers in *T. hemsleyanum* (ug/g) (continued)**

|  | Apigenin | Apigenin-7G | Kaempferol | Astragaline | Luteolin | Luteoloside |
| --- | --- | --- | --- | --- | --- | --- |
| CK | 0.023±0.001de | 0±0a | 0.366±0.058h | 7.484±1.501hi | 0±0a | 0±0a |
| UV-A (15min) | 0.02±0.002f | 0±0a | 1.149±0.06ef | 48.586±2.518f | 0±0a | 0±0a |
| UV-A (30min) | 0.02±0.003ef | 0±0a | 0.22±0.006h | 21.313±1.286g | 0±0a | 0±0a |
| UV-A (1h) | 0.031±0.004ab | 0±0a | 0.249±0.009h | 0.304±0.007i | 0±0a | 0±0a |
| UV-A (2h) | 0.022±0.002de | 0±0a | 0.332±0.017h | 0.647±0.059i | 0±0a | 0±0a |
| UV-A (3h) | 0.024±0.001de | 0±0a | 0.351±0.009h | 104.076±4.031d | 0±0a | 0±0a |
| UV-A (5h) | 0.02±0.001ef | 0±0a | 3.853±0.431b | 131.164±8.006c | 0±0a | 0±0a |
| UV-B (15min) | 0.024±0.003cd | 0±0a | 0.888±0.195fg | 51.695±7.611f | 0±0a | 0±0a |
| UV-B (30min) | 0.027±0.001bc | 0±0a | 2.221±0.266c | 163.377±12.699a | 0±0a | 0±0a |
| UV-B (1h) | 0.028±0.006ab | 0±0a | 1.22±0.085ef | 63.443±2.626e | 0±0a | 0±0a |
| UV-B (2h) | 0.026±0.006cd | 0±0a | 1.753±0.36d | 142.512±1.564b | 0±0a | 0±0a |
| UV-B (3h) | 0.027±0.001bc | 0±0a | 1.378±0.076de | 0.507±0.009i | 0±0a | 0±0a |
| UV-B (5h) | 0.035±0a | 0±0a | 1.193±0.044ef | 63.321±1.829e | 0±0a | 0±0a |
| UV-C (15min) | 0.028±0.004ab | 0±0a | 0.127±0.009h | 2.368±0.009i | 0±0a | 0±0a |
| UV-C (30min) | 0.024±0.005cd | 0±0a | 5.848±0.088a | 50.814±1.194f | 0±0a | 0±0a |
| UV-C (1h) | 0.028±0.002bc | 0±0a | 0.482±0.008gh | 6.23±0.083i | 0±0a | 0±0a |
| UV-C (2h) | 0.034±0.001ab | 0±0a | 2.494±0.191c | 20.981±1.21g | 0±0a | 0±0a |
| UV-C (3h) | 0.022±0.002de | 0±0a | 0.291±0.016h | 5.663±0.651i | 0±0a | 0±0a |
| UV-C (5h) | 0.023±0.004de | 0±0a | 1.101±0.047ef | 17.668±0.72gh | 0±0a | 0±0a |

**Supplementary Table 4** **Effect of UV treatment on flavonoid monomers of root tubers in *T. hemsleyanum* (ug/g) (continued)**

|  | Vitexin | Hesperidin | Quercitrin | Isoquercitrin | Rutin | Eriodictyol |
| --- | --- | --- | --- | --- | --- | --- |
| CK | 49.643±0.89hi | 0±0d | 0.02±0g | 1.115±0.19hi | 0±0d | 0.023±0.002de |
| UV-A (15min) | 1423.629±10.203a | 0±0d | 0.024±0de | 6.165±0.805f | 0.007±0.001d | 0.014±0.002gh |
| UV-A (30min) | 114.911±2.602fg | 0±0d | 0.024±0.001d | 3.708±0.024g | 0.063±0.004d | 0.009±0.001j |
| UV-A (1h) | 102.039±2.694gh | 0±0d | 0.021±0fg | 0±0j | 0.14±0.008d | 0.01±0ij |
| UV-A (2h) | 561.743±7.363c | 0±0d | 0.022±0ef | 1.618±0.008hi | 1.055±0.022b | 0.03±0.001c |
| UV-A (3h) | 114.128±9.003fg | 0±0d | 0.043±0.001a | 37.962±1.394a | 0.011±0.001d | 0.009±0.001j |
| UV-A (5h) | 79.921±5.94gh | 0±0d | 0.03±0bc | 21.228±0.466b | 0.006±0.001d | 0.054±0.002b |
| UV-B (15min) | 126.185±5.361fg | 0±0d | 0.028±0.003c | 8.263±0.928e | 0.073±0.012d | 0.016±0.002fg |
| UV-B (30min) | 21.892±2.107ij | 0±0d | 0.021±0.001ef | 13.637±1.162d | 0.099±0.024d | 0.013±0.001hi |
| UV-B (1h) | 95.22±4.792gh | 0±0d | 0.032±0b | 13.96±0.655d | 0.044±0.007d | 0.019±0ef |
| UV-B (2h) | 172.338±7.002ef | 0±0d | 0.032±0bc | 21.479±0.661b | 0.016±0.002d | 0.072±0.003a |
| UV-B (3h) | 204.533±2.486e | 0±0d | 0.021±0fg | 0.201±0.014ij | 0.295±0.014d | 0.013±0.001hi |
| UV-B (5h) | 648.619±109.751b | 0±0d | 0.024±0.001de | 15.858±0.121c | 5.375±0.827a | 0.015±0.003gh |
| UV-C (15min) | 70.739±1.762gh | 0.295±0.015a | 0.022±0.001de | 0.418±0.029ij | 0±0d | 0±0k |
| UV-C (30min) | 68.679±2.963gh | 0±0d | 0.024±0.002de | 15.916±0.54c | 0.416±0.006d | 0.008±0.001j |
| UV-C (1h) | 280.261±4.111d | 0±0d | 0±0h | 2.155±0.048h | 0.931±0.092bc | 0.007±0.001j |
| UV-C (2h) | 683.912±7.982b | 0.174±0.009b | 0.024±0.001de | 7.825±0.174e | 0.019±0.001d | 0.023±0.002d |
| UV-C (3h) | 3.639±0.37k | 0.041±0.007c | 0±0h | 0.889±0.138hi | 0.322±0.003d | 0±0k |
| UV-C (5h) | 7.237±0.649jk | 0±0d | 0.023±0.001de | 5.154±0.431fg | 0.466±0.06cd | 0±0k |

**Supplementary Table 4** **Effect of UV treatment on flavonoid monomers of root tubers in *T. hemsleyanum* (ug/g) (continued)**

|  | Lonicerin | Diosmetin | Diosmetin-7G | Myricetin | Baicalin |
| --- | --- | --- | --- | --- | --- |
| CK | 0±0e | 0.004±0b | 0±0a | 0.012±0.001ij | 0±0e |
| UV-A (15min) | 0±0e | 0.001±0e | 0±0a | 0.016±0hi | 0.035±0.001cd |
| UV-A (30min) | 0±0e | 0.002±0.001de | 0±0a | 0.01±0j | 0±0e |
| UV-A (1h) | 0.019±0.006e | 0.002±0e | 0±0a | 0.013±0.001ij | 0.037±0.001cd |
| UV-A (2h) | 0.07±0.02e | 0.003±0c | 0±0a | 0.013±0.001ij | 0±0e |
| UV-A (3h) | 0.016±0.002e | 0.002±0e | 0±0a | 0.084±0.002d | 0±0e |
| UV-A (5h) | 0.024±0.005e | 0.001±0e | 0±0a | 0.119±0.008b | 0±0e |
| UV-B (15min) | 0.02±0.002e | 0.001±0e | 0±0a | 0.013±0ij | 0.036±0.007cd |
| UV-B (30min) | 0.305±0.048de | 0.002±0d | 0±0a | 0.023±0.002h | 0.046±0.002cd |
| UV-B (1h) | 0.096±0.002e | 0.002±0e | 0±0a | 0.053±0.004e | 0.06±0.001c |
| UV-B (2h) | 0.033±0.006e | 0.002±0e | 0±0a | 0.022±0.001h | 0.035±0.003d |
| UV-B (3h) | 0.052±0.009e | 0.002±0d | 0±0a | 0.012±0.001ij | 0.032±0.003d |
| UV-B (5h) | 7.572±1.102a | 0.001±0e | 0±0a | 0.045±0.003f | 0.029±0.006d |
| UV-C (15min) | 0±0e | 0.001±0f | 0±0a | 0.037±0.006g | 0.035±0.001cd |
| UV-C (30min) | 0.824±0.026cd | 0.001±0e | 0±0a | 0.134±0.005a | 0.328±0.042a |
| UV-C (1h) | 1.495±0.088b | 0.001±0f | 0±0a | 0.022±0.002h | 0.089±0.005b |
| UV-C (2h) | 0.022±0.007e | 0.006±0a | 0±0a | 0.06±0.002e | 0.06±0.003c |
| UV-C (3h) | 0.782±0.077cd | 0±0f | 0±0a | 0.018±0.002hi | 0.038±0.003cd |
| UV-C (5h) | 1.306±0.097bc | 0.002±0de | 0±0a | 0.119±0.002c | 0.044±0.001cd |

# Supplementary Methods

## Supplementary Method 1

**Determination of total flavonoid content**

**Determination of sample content**

A total of 1.0 g leaves (L) and root tubers (R) were weighed, ground until pulp, added with 50 mL 75% ethanol solution, poured into a 150 mL conical bottle, and subjected to ultrasonic extraction. The ultrasonic conditions were as follows: 55 °C, 80 W, 40 min, and two times experimentation. After the extraction, we collected the filtrate in a constant-volume 50 mL volumetric flask, accurately obtained 4 mL filtrate in a 25 mL volumetric flask, added 75% ethanol until 6 mL volume, and set aside the mixture for 6 min. Then, 10% aluminum nitrate (1 mL) was added, and the mixture was shaken well and set aside for 6 min. Next, 10% sodium hydroxide 10 mL and 75% ethanol were added until the scale line, and the mixture was shaken well and stored for 15 min. The total flavonoid content in the L and R of *T. hemsleyanum* were determined by a UV-VIS spectrophotometer (UNICO 3802, America), and the methodology was investigated.

**Investigation of linear relation**

A total of 0.8 mg/mL rutin standard solution was prepared, and 0.5, 1, 1.5, 2, and 2.5 mL rutin standard solution were collected in 25 mL volumetric flasks separately and added with 75% ethanol until 6 mL volume, followed by the addition of 10% aluminum nitrate 1 mL. The mixtures were shaken well and set aside for 6 min. The 10% sodium hydroxide was added until 10 mL volume, followed by 75% ethanol to the scale line, and the mixtures were shaken well and set aside for 15 min. The absorbance was determined at 510 nm, and the standard curve was drawn.

**Stability examines**

The absorbance value was measured at 0, 5, 10, 15, 20, 25, and 30 min, and the relative standard deviation (RSD) was calculated.

**Repeatability survey**

The absorbance value of five samples was determined, and the RSD was calculated.

**Precision survey**

The absorbance value of the standard solution was determined five times, and the RSD value was calculated.

## Supplementary Method 2

The extraction method of crude enzyme solution was as follows: A total of 0.5 g fresh L and R were ground with 9 mL buffer solution into a homogenate and centrifuged at 10,000 r/min for 15 min at 4 °C. The supernatant was used as the extract.

CAT concentration was determined as follows: The 200 µL enzyme extract and 2.98 mL reaction solution (100 mL 0.2 mol/L pH = 7.8 phosphoric buffer added 0.2 mL 30% H_2_O_2_ ) were mixed. The change in absorbance within 3 min was measured at 240 nm.

POD concentration was determined as follows: Exactly 400 µL enzyme extract and 2.96 mL reaction solution (100 mL 50 mmol/L pH = 7.4 phosphate buffer, 1 mL 30% H_2_O_2_; 0.2 mL guaiacol) were mixed. The change in absorbance was measured at 470 nm within 3 min.

SOD concentration was determined as follows: Several test tubes were obtained, with tuber 1, tuber 2, and other tubers as samples. The solution was added in accordance with Supplementary Table 5. After mixing, tuber 1 was stored in the dark. Tuber 2 and the sample tubers were exposed to light (4000 Lx) for 20 min. After the reaction, tuber 1 was adjusted to zero, tuber 2 was the CK, and the absorbance was determined at 560 nm.

**Supplementary Table 5 Method for determination of SOD enzyme activity**

| **Reagent name(mL)** | **Tubers for samples** | **Tuber 1** | **Tuber 2** |
| --- | --- | --- | --- |
| 50mmol/L [phosphate](javascript:;) [buffer](javascript:;) | 5.0 | 5.0 | 5.0 |
| 130mmol/L Met solution | 0.3 | 0.3 | 0.3 |
| 750μmol/L NBT solution | 0.3 | 0.3 | 0.3 |
| 100μmol/L EDTA-Na_2_ | 1.0 | 1.0 | 1.0 |
| [distilled](javascript:;) [water](javascript:;) | 2.0 | 2.0 | 2.0 |
| 20μmol/L [riboflavin](javascript:;) | 0.3 | 0.3 | 0.3 |
| [sample](javascript:;) | 0.3(enzyme liquid) | 0.3([buffer](javascript:;) [solution](javascript:;)) | 0.3([buffer](javascript:;) [solution](javascript:;)) |

The MDA content was determined as follows: A total of 2 mL extracting solution was added with 2 mL 0.6% TBA (configured with 10% TCA) and heated in a water bath for 30 min at 95 ℃. After cooling for 20 min on ice, the precipitate was removed by centrifugation at 10,000 rpm for 15 min, and its absorbance was determined at 450, 532, and 600 nm.

## Supplementary Method 3

**Determination of free amino acid content**

Standard curve determination: A total of 5 µg/mL standard leucine solutions (0, 0.2, 0.4, 0.6, 0.8, and 1.0 mL) were obtained, diluted with distilled water to 2.0 mL, added with 3.0 mL hydrating ninhydrin reagent, and finally added with 0.1% ascorbic acid 0.1 mL. The mixtures were heated by boiling water bath for 15 min and cooled, and the absorbance was measured at 570 nm.

Sample determination: The L and R under different treatments were collected, weighed (0.5 g) accurately, added with 5.0 mL 10% acetic acid solution, ground into homogenates in a mortar, diluted with distilled water to 30 mL, and filtered. Then, 0.5 mL sample solution, 1.5 mL distilled water, 3.0 mL hydrinhydrin reagent, and 0.1 mL 0.1% ascorbic acid were successively added, and the mixtures were heated in boiling water bath for 15 min and cooled. Absorbance was determined at 570 nm.

**Determination of soluble protein content**

Standard curve determination: A total of 100 μg/mL bovine serum protein of different volumes were prepared (0, 0.2, 0.4, 0.6, 0.8, and 1.0 mL), added with distilled water to 1.0 mL followed by 5.0 mL Coomasie bright blue reagent, shaken well, and set aside for 2 min. Absorbance was determined at 595 nm.

Sample determination: The L and R were collected after different treatments. Then, 0.5 g of these plant parts were weighed and ground into homogenate with 5.0 mL distilled water in a mortar and centrifuged at 3000 r/min for 10 min to obtain the supernatant. Next, 1.0 mL sample was absorbed, added with 5.0 mL Coomasie bright blue reagent, and shaken well. The optical density was measured at 595 nm after 2 min.

**Determination of soluble sugar content**

Standard curve measurement: Standard glucose solutions (0, 40, 80, 120, 160, and 200 µg/mL) were prepared. Then, 0.5 mL anthranone reagent was added to each tube followed by the slow addition of 5.0 mL concentrated sulfuric acid in ice water bath. The mixtures were shaken well, heated for 10 min in a water bath, and then cooled to room temperature. The optical density was measured at 620 nm.

Sample determination: A total of 0.5 g L and R were weighed after different treatments, ground into homogenate in a mortar with 5.0 mL 85% ethanol solution, and heated with water bath (50 °C) for 30 min. The extracting solution was filtrated twice and merged. Ethanol was steamed by rotary evaporator and added with distilled water into a 25 mL volumetric flask. Then, 1.0 mL sample solution was collected and added with 1.0 mL distilled water and 0.5 mL anthrone reagent. Next, 5.0 mL concentrated sulfuric acid was slowly added in ice water bath. Then, the mixtures were shaken well, heated for 10 min in a water bath, removed, and cooled to room temperature. The optical density was measured at 620 nm.
